# Multitechnique characterization of secondary minerals near HI-SEAS, Hawaii, as Martian subsurface analogues

**DOI:** 10.1038/s41598-023-48923-7

**Published:** 2023-12-18

**Authors:** Sebastian J. Mulder, Frank J. A. van Ruitenbeek, Bernard H. Foing, Mónica Sánchez-Román

**Affiliations:** 1 Energy and Sustainability Research Institute Groningen, Faculty of Science and Engineering, University of Groningen, Nijenborgh 6, 9746 AG Groningen, The Netherlands; 2 Earth Sciences Department, Science Faculty, Vrije Universiteit Amsterdam, De Boelelaan 1085, 1081 HV Amsterdam, The Netherlands; 3 Department of Applied Earth Sciences, Faculty of Geo-Information Science and Earth Observation, University of Twente, Drienerlolaan 5, 7500 AE Enschede, The Netherlands; 4 LUNEX/ILEWG EuroMoonMars & Leiden Observatory, Universiteit Leiden, Niels Bohrweg 2, 2333 CA Leiden, The Netherlands

**Keywords:** Mineralogy, Petrology, Mineralogy

## Abstract

Secondary minerals in lava tubes on Earth provide valuable insight into subsurface processes and the preservation of biosignatures on Mars. Inside lava tubes near the Hawaii-Space Exploration and Analog Simulation (HI-SEAS) habitat on the northeast flank of Mauna Loa, Hawaii, a variety of secondary deposits with distinct morphologies were observed consisting of mainly sodium sulphate powders, gypsum crystalline crusts, and small coralloid speleothems that comprise opal and calcite layers. These secondary deposits formed as a result of hydrological processes shortly after the formation and cooling of the lava tubes and are preserved over long periods of time in relatively dry conditions. The coralloid speleothem layers are likely related to wet and dry periods in which opal and calcite precipitates in cycles. Potential biosignatures seem to have been preserved in the form of porous stromatolite-like layers within the coralloid speleothems. Similar secondary deposits and lava tubes have been observed abundantly on the Martian surface suggesting similar formation mechanisms compared to this study. The origin of secondary minerals from tholeiitic basalts together with potential evidence for microbial processes make the lava tubes near HI-SEAS a relevant analog for Martian surface and subsurface environments.

## Introduction

In the last two decades, there is an increasing interest in secondary minerals found in lava tubes as they can serve as a proxy to determine the speleogenetic history of the lava tubes and can preserve potential biosignatures^1,2^. A variety of secondary minerals have been observed in many of these lava tubes such as on Hawaii, the Azores, Canary Islands, Easter Island, Iceland, USA, and Japan^3–5^. The interior of lava tubes provide stable subsurface conditions for the growth of secondary minerals by physiochemical processes, however, the process itself and the type of mineral phases that are left behind are not fully understood^6,7^. Furthermore, it is often uncertain if the morphological features within caves, such as stromatolite and/or microbial mat like-layers, are formed by abiotic or biotic processes, although previous studies have concluded that the presence of microbes likely has an impact on the evolution and preservation of stromatolite and/or microbial mat in lava tubes^1,7^.

Lava tubes are observed on Mars as well, where the lower gravitational force could make lava tubes more resistant to collapse, which could lead to the formation and preservation of secondary minerals^8^. Additionally, these lava tubes provide stable conditions against the hostile environment (e.g., fluctuating temperatures, high doses of radiation, dust storms) residing on the Martian surface. These stable conditions together with the presence of water could have provided habitable environments for alien life. Consequently, secondary mineralization in lava tubes on Earth can serve as an analogue to the formation of authigenic minerals in lava tubes on Mars from a geological and astrobiological perspective.

Previous studies on the Big Island of Hawaii focused on secondary mineralization in old lava tubes in coastal areas characterized by wet and ambient temperature climates in which microbial mats and organic ooze are abundantly present and to a lesser extent at higher elevations where there is a relatively drier climate^4,9^. Secondary minerals were observed in relatively young lava tubes near the Hawaii-Space Exploration and Analog Simulation (HI-SEAS) habitat on Mauna Loa, Hawaii, during the EMMIHS1 2019 EuroMoonMars campaign. The aim of this study was to analyse the secondary minerals in these lava tubes to understand the characteristics and formation processes of the mineral precipitates in relatively dry conditions at a high altitude as an analogue for the presence of such minerals in lava tubes on Mars. For this, a multidisciplinary approach has been used combining field observations with mineralogical, geochemical, microscopic and infrared spectroscopy analyses. The observed secondary minerals consist of thenardite/mirabilite, gypsum, and opal and calcite layered speleothems with minor traces of monohydrocalcite. Similar deposits have been found in cryogenic settings in other lava tubes on Mauna Loa close to the research area^10^. These deposits consist of gypsum, SiO_2_ and cryogenic calcite. The aim of this study was to identify the different secondary mineral morphologies and mineralogy in lava tubes at high altitudes in relatively dry conditions as analogue for Mars and to acquire high spatial and spectral resolution images to mimic similar analytical applications used for future Mars missions.

## Geographical and geological setting

**Figure 1:**
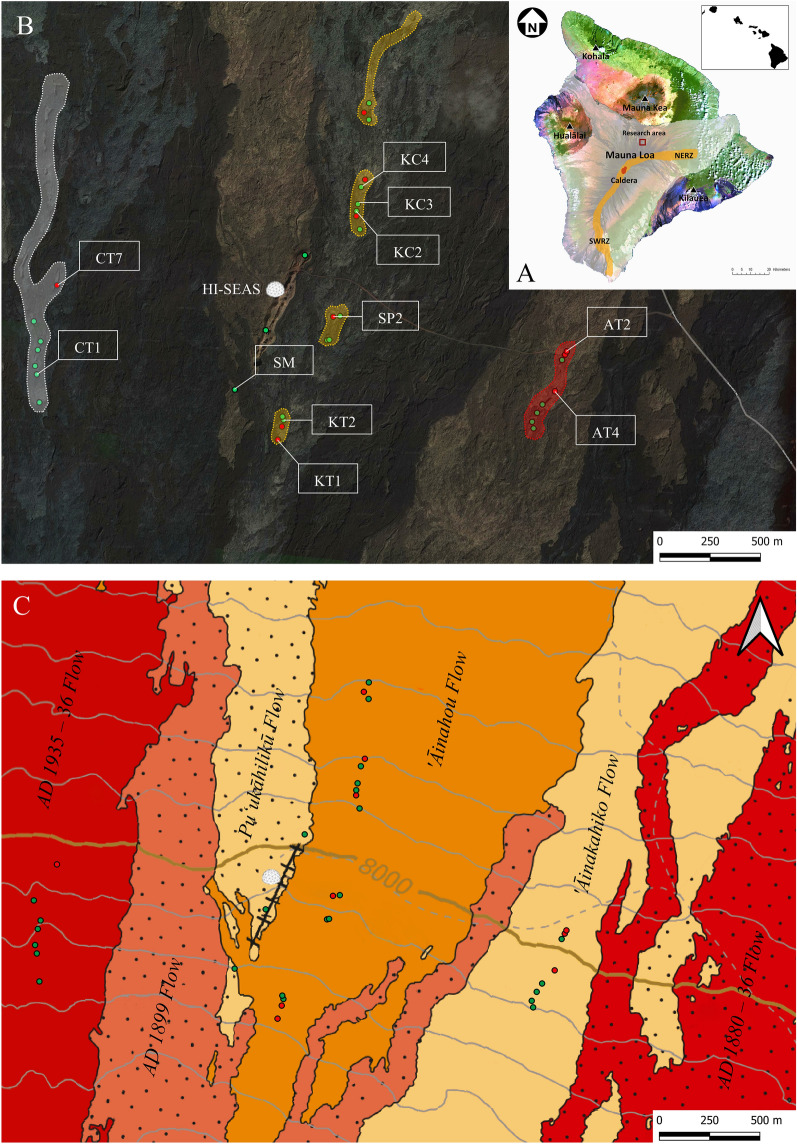
Maps showing the location of Hawaii and the research area. (**A**) Landsat image of the Big Island, Hawaii, and the five volcanoes of which Mauna Loa is the largest (white zone). Eruptions derive from the caldera, the Northeast Rift Zone (NERZ) and the Southwest Rift Zone (SWRZ). The research area lies on the north-eastern flank of Mauna Loa (red square). The image is from the National Oceanic and Atmospheric Administration. (**B**) An overview of the research area near the HI-SEAS habitat with the lava tube systems, mapped skylights (green dots), mapped lava tube entrances (red dots) and sample locations (digitized on Google Hybrid map layer). 'Āinakahiko Tube (red), 'Āinahou Tube (orange) and Columbus Tube (white). The map has been produced with the open source software Geographic Information System (GIS) 3.12 (https://www.qgis.org/en/site/). (**C**) Geological map of the lava flows in the research area (modified from Trusdell and Zoeller, 2017). The Black line adjacent to the habitat represents the fissure ridge.

The HI-SEAS habitat is on the northeast flank of Mauna Loa, Big Island of Hawaii, one of five shield-volcanoes of which the island is composed and the largest active volcano on Earth (Fig. 1A). The volcano is approximately 4200 m high and covers roughly 51% of the island’s surface area, which consists of ~ 500 classified lava flows, mainly tholeiitic basalts, that formed over 30 kyr^11–13^. The eruptions derive mainly from two active elongated rift zones that originate from the summit caldera, Moku‘āweoweo, extending to the northeast and southwest^12^.

The research area near the HI-SEAS habitat lies north of the Northeast Rift Zone (NERZ) at an altitude of ~ 2500 m where it is relatively dry with annual precipitation rates of 508–762 mm and annual temperatures between 3 and 12 °C, according to Mauna Loa Observatory (MLO) data. The research area consists of several lava flows that emerged from the NERZ or nearby fissures (Fig. 1). Almost all of these lava flows contain partially collapsed elongated lava tube systems that have an SSW to NNE direction. Radiocarbon dating of mainly carbonized plant debris samples collected from the lava flows show that the age of the *'*Āinakahiko Flow is dated at 1880 ± 200 year B.P., Pu‘ukāhilikū Flow at 1838 ± 94 year B.P. and 'Āinahou Flow at 410 ± 60 year B.P (Fig. 1C)^14^. These ages are calibrated to calendar years using the CALIB 4.0 Radiocarbon Calibration Program^14,15^.

## Results

### Secondary mineral morphology and identification

28 samples were taken from three lava tube systems that are all located in a different lava flows (Fig. 1). From these 28 samples, 14 samples that were taken deep inside the lava tubes were further analysed. A detailed description of the three lava tubes can be found in the Material and methods section. A wide variety of secondary mineral morphologies have been observed in the lava tubes. Therefore, the secondary minerals are classified on the basis of specimen morphology and mineral composition (Supplementary Table S1). Three morphology types are recognized and described in terms of field, microscopic and mineralogical observations: (1) white powders, (2) crystalline crusts, and (3) coralloid speleothems. Table 1 shows the major elemental compositions of the basaltic rocks that are representative as host rock on which the secondary mineral samples were formed. Rock samples contain a relatively high amount of MgO and CaO, being typical tholeiitic with minor abundances of alkaline material (Table 1). Despite the age difference between the lava flows and the amount of weathering each lava flow underwent, there is little difference between the various samples in terms of composition.

**Table 1:** Geochemistry of the basaltic host rocks as determined by X-ray fluorescence.

Sample	Lava flow	Fe_2_O_3_ (%)	MnO (%)	TiO_2_ (%)	CaO (%)	K_2_O (%)	P_2_O_5_ (%)	SiO_2_ (%)	Al_2_O_3_ (%)	MgO (%)	Na_2_O (%)	BaO (%)
SM-A	Pu‘ukāhilikū	12.46	0.17	2.15	10.25	0.40	0.24	50.84	13.43	6.37	2.59	0.01
KC3-A	'Āinahou	11.70	0.16	2.03	10.31	0.44	0.25	51.18	13.53	6.95	1.87	0.01
KC4-A	'Āinahou	11.48	0.16	1.99	10.25	0.43	0.24	50.47	13.37	6.84	2.49	0.01
SP2-A	'Āinahou	11.55	0.16	2.01	10.26	0.40	0.24	50.69	13.43	6.79	2.04	0.01
CT1-A	A.D. 1935–36	12.12	0.17	2.08	10.49	0.37	0.22	51.51	13.64	7.01	2.29	0.01

### Sulphate powders

**Figure 2:**
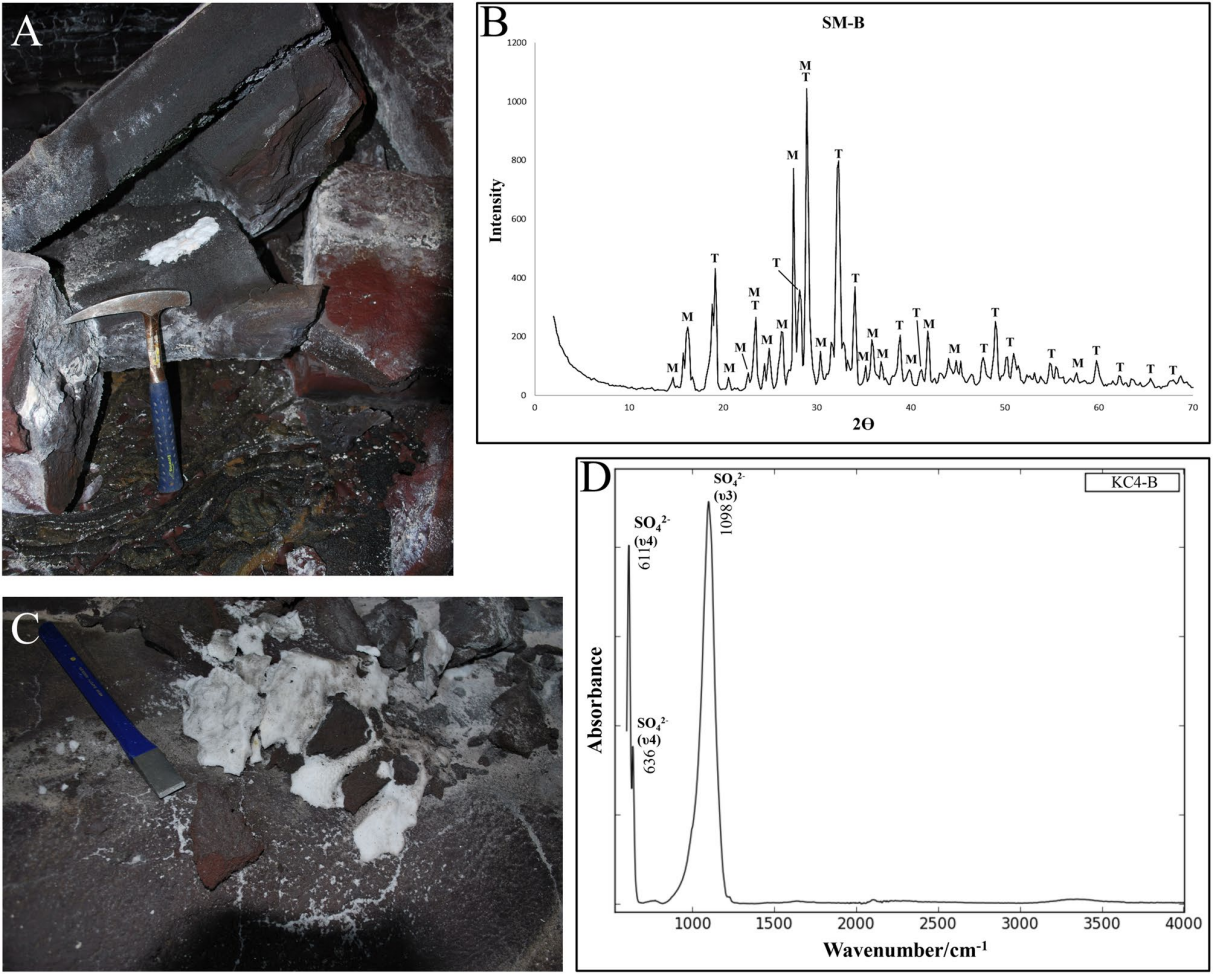
Characteristics and composition of the sulphate powders and SWIR spectra of the secondary minerals. (**A**) Morphology of the white powder accumulates as observed in the field (KC2). (**B**) XRD pattern of the white deposit (SM-B) consisting out of a mix of thenardite (T) and mirabilite (M). (**C**) White sulphate powder deposit with a crystalline salt like structure (KC4). (**D**) FTIR spectrum representative for the sulphate powders consisting of pure sulphate compounds.

**Figure 3:**
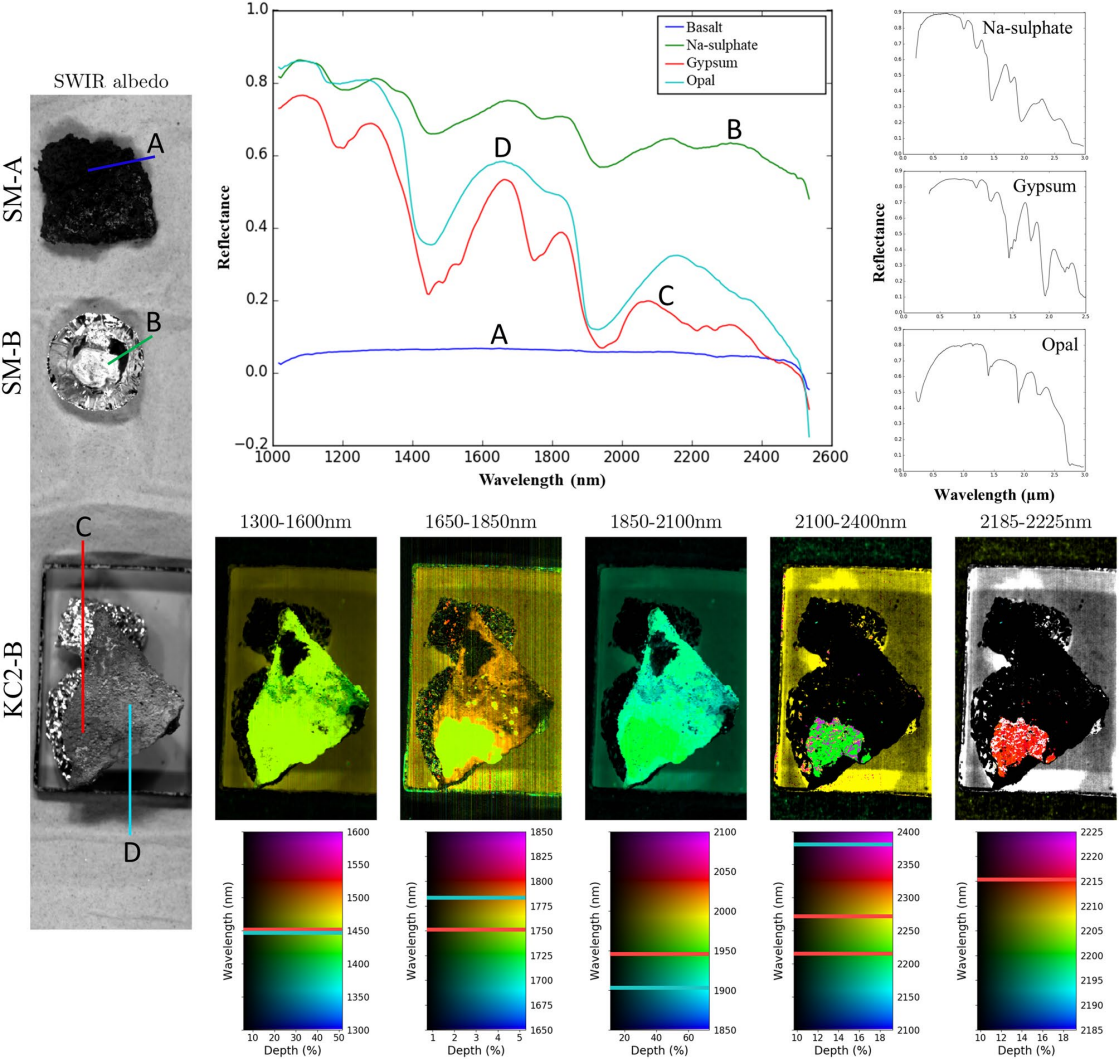
Individual short wave reflectance spectra of the different secondary minerals found in the lava tube systems, wavelength maps of sample KC2-B, and black spectra from the USGS spectral library (Mirabilite—GDS150, Gypsum—HS333.3B, Opal—TM8896). The coloured lines in the albedo image show the location from which the spectra were obtained in the sample and correspond with their respective colour of the spectra in the figure. The lines in the wavelength map legends show the deepest absorption features for gypsum (red) and opal (cyan). Note that the aluminium foil that supports the rock samples shows pixels with absorption features within the wavelength maps.

The white powder is present in all three lava tube systems where it usually accumulates in cracks or on the cave floors as small dry piles (diameter 5–20 cm) with basaltic rock directly overhead (Fig. 2A). This powder is identified as sodium sulphate, thenardite (Na_2_SO_4_), or in its hydrous phase mirabilite (Na_2_SO_4_·10H_2_O) (Fig. 2B, C). Mirabilite was only observed at two locations (Fig. 1B: SP2, KC4). However, no observations have been made whether or not the type of mineral morphology was location dependent. Fourier-Transform Infrared (FTIR) spectra of all the white powders show strong absorbance peaks corresponding to SO_4_^2−^ bonds for the υ3 stretch at ~ 1098 cm^−1^ and the υ4 stretch at ~ 611 and ~ 637 cm^−1^, respectively (Fig. 2D). Absorbance peaks of OH^−^ bonds are absent in FTIR spectra as the powder samples were heated prior to analysis during which the sulphate powders change to its anhydrous constituent. In general, Short-Wave Infrared (SWIR) spectra of the sulphate powders have high reflectance and show multiple strong absorption bands at ~ 1192, ~ 1452, ~ 1948 and ~ 2200 nm related to H_2_O bonds, which are also observed in the other secondary mineral samples of this study and their respective wavelength maps (Fig. 3).

### Crystalline crusts

**Figure 4:**
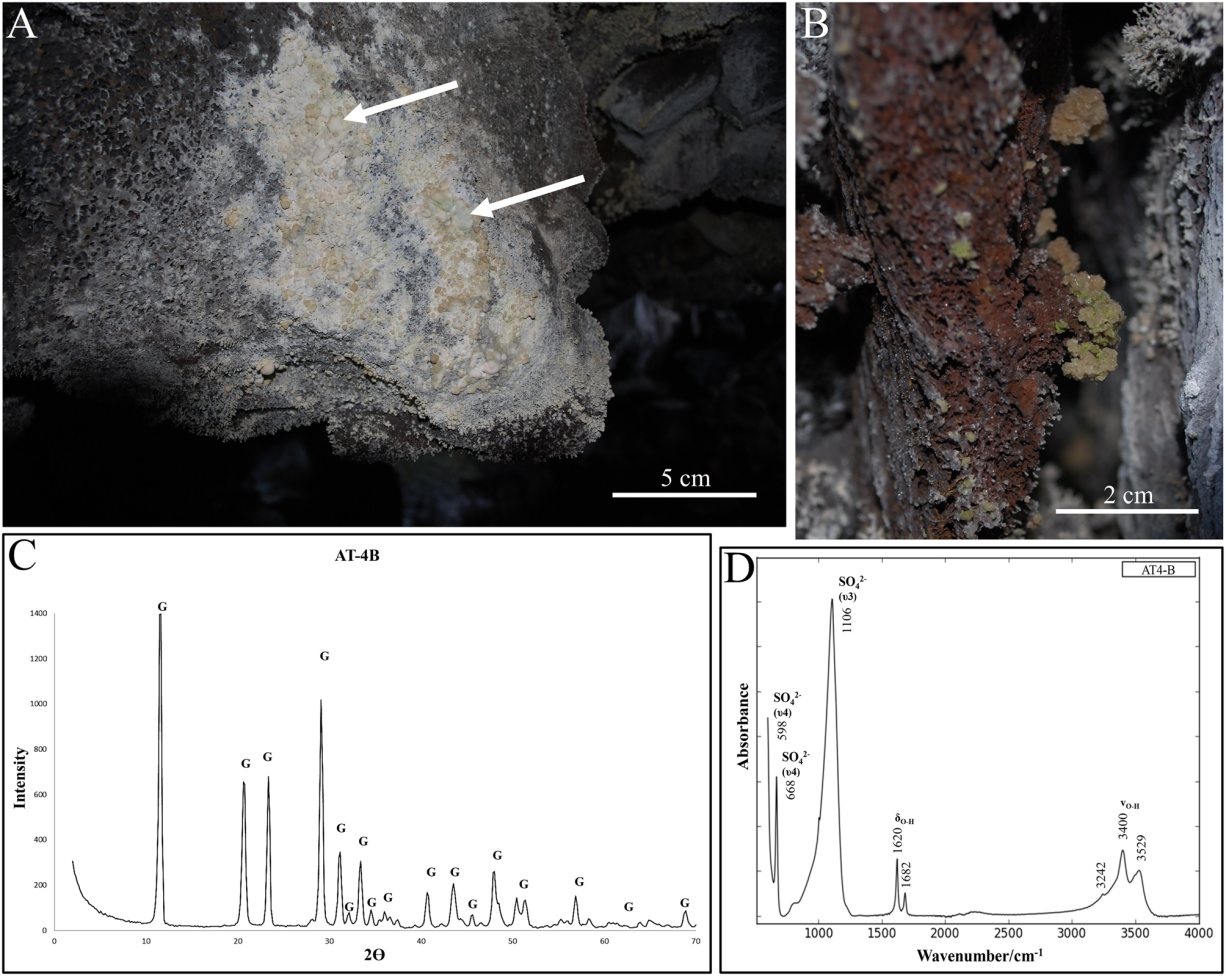
Characteristics and composition of the crystalline crusts. (**A**) Patches of white to green botryoidal crystalline crusts of gypsum, which is often observed on the cave walls and ceilings in all lava tube systems. White arrows indicate Gypsum puffballs. (**B**) Dark green with orange crystalline gypsum globules on the surface of basaltic rock. (**C**) XRD pattern of crystalline crust, consisting of gypsum (G). (**D**) FTIR spectrum of the crystalline crusts of which the vibration modes are typical for gypsum.

The crystalline crusts are identified as gypsum (CaSO_4_·2H_2_O) and formed as thin (3 mm) small patches on the surfaces of basaltic rock either on the ceiling or walls, where it is often found together with the coralloid speleothems (Fig. 4A, C). These crystallized crusts usually have a dark to light green botryoidal morphology sometimes intercalated with small puffballs although some white translucent microcrystalline deposits have also been observed. At *'*Āinakahiko Tube, the secondary mineral has grown as crystalline globules on rough surfaces in cracks with sometimes brown-orange glossy colour, which were also sometimes present as small stalactites between the coralloid speleothems (Fig. 4B). FTIR spectra show the same υ3 (~ 1106 cm^−1^) and υ4 (~ 596 and ~ 668 cm^−1^) SO_4_^2−^ stretching bands as thenardite with absorption peaks of incorporated H_2_O at ~ 1620, ~ 3400, and ~ 3525 cm^−1^ that corresponds to bending and stretching vibrations of OH (Fig. 4D). The SWIR spectra show the deep absorption feature of SO_4_^2−^ at ~ 1750 nm that is typical for gypsum and H_2_O absorption bonds at ~ 1193, ~ 1443 and ~ 1945 nm (Fig. 3). Gypsum distinguishes itself from the coralloid speleothems in the wavelength maps at higher wavelength intervals where gypsum has deep absorption features at 2215 and 2268 nm opposed to the other secondary mineral, while wavelength maps at lower wavelength intervals show deep absorption features in a close colour range due to the deep absorption features of H_2_O that are present in both secondary minerals (Fig. 3). Black pixels indicate that absorption features are absent or shallow within the respective wavelength range.

### Coralloid speleothems

**Figure 5:**
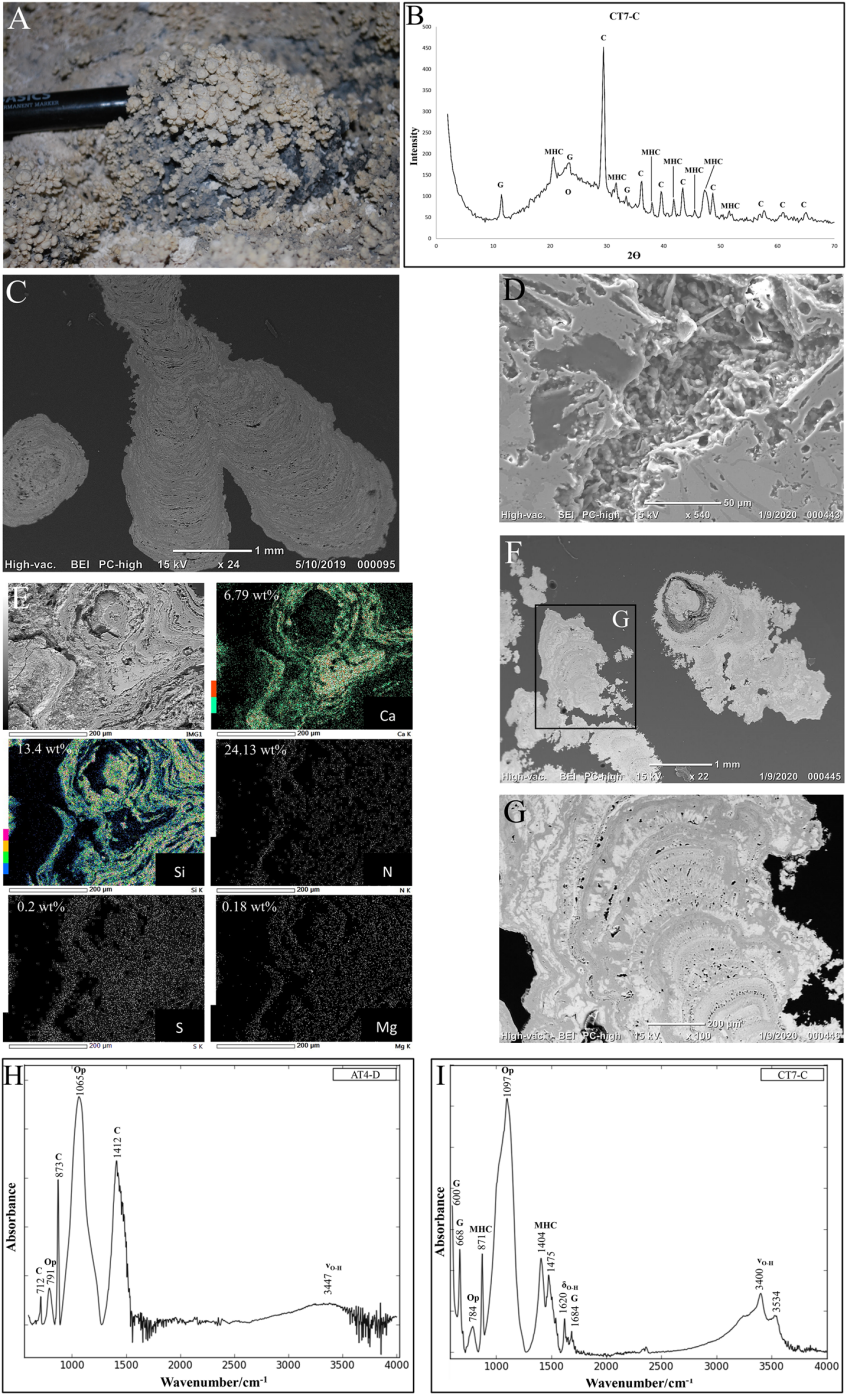
Morphology and geochemical composition of the coralloid speleothems. (**A**) Coralloid speleothems on the floor at sample location AT4. (**B**) XRD of sample CT7-C with peaks corresponding to mainly calcite (C) and opal (O) with minor abundances of gypsum (G) and monohydrocalcite (MHC). (**C**) SEM image of a coralloid speleothem (AT4-D) consisting of alternating layers of calcite (light) and opal (dark) in longitudinal section (right) as well as in cross-section (left). (**D**) Botryoidal silica nanospheres in the vugs of sample KC2-B. (**E**) Elemental map and quantitative analysis of sample KC2-B. Elemental mass can be found in Supplementary Fig. S3. Abbreviations stand for calcite (Ca), silica (Si), nitrogen (N), sulphide (S), and magnesium (Mg). (**F**) SEM image of a coralloid speleothem (KT2-B) with a high porosity around the core structure. (**G**) SEM images of acicular needle like crystals in the calcite layers of a coralloid speleothem (KT2-B). (**H**) FTIR spectrum of sample AT4-D consisting of opal (Op) and calcite (C) compounds together with vibration band corresponding to water (OH). (**I**) FTIR spectrum of sample CT7-C consisting opal, monohydrocalcite (MHC) and gypsum (G) compounds together with vibration bands corresponding to water.

The coralloid speleothems have generally an irregular branching botryoidal morphology in various colours from white, green to white yellow that seems to evolve from small thin stalactites to well-developed broad globular speleothems (Fig. 5A). These speleothems range from ~ 3 to ~ 9 mm in length and from 1 to 4 mm in diameter. In *'*Āinakahiko Tube, some small patches (~ 4 cm^2^) of puffy botryoidal coralloid speleothems are observed that exist of loose powdery material. Another form of these coralloid speleothems is the aforementioned small thin (1–2 mm) light green botryoidal hemispherical patches that cover the surface of lava stalactites (Supplementary Fig. S2E). At one of the lava tube entrances (AT2), lithified lichen is observed on the ceiling next to active lichen (Supplementary Fig. S2B). An integrated approach of the different analytical techniques shows that the coralloid speleothems comprises mainly of opal-A (SiO_2_·nH_2_O) and calcite (CaCO_3_), and that metal hydroxide bonds are scarce in the samples, indicating that phyllosilicates and other clay minerals are not abundantly present in these secondary minerals. X-Ray Diffraction (XRD) patterns of the coralloid speleothems show mainly a mixture of calcite together with smaller abundances of monohydrocalcite (CaCO_3_·H_2_O), gypsum, and opal-A (Fig. 5B). Scanning Electron Microscope (SEM) images of the coralloid speleothems reveal compact alternating concentric layers of opal-A and calcite, which show similarities to stromatolites (Fig. 5C). The coralloid speleothems contain many vugs that are mainly parallel to the layering in which the surface has a botryoidal morphology consisting of silica microspheres with the presence of microbial filaments (Fig. 5D). Some samples display a core structure around which the layering is much more porous than the rest of the internal structure (Fig. 5F). Additionally, the internal structure of some of the samples contains acicular carbonate crystal needles (~ 80 μm) that are perpendicular to the layering and has a dendritic morphology (Fig. 5G). Elemental mapping shows that there are minor amounts of sulphate and magnesium present within some speleothems and a high amount of nitrogen (± 25 wt%) in one of the samples (Fig. 5E).

The secondary minerals were kept intact on the basaltic surfaces for SWIR analyses prior to powdering in order to acquire hyperspectral images of the mineral distribution on the basaltic surfaces of the samples to mimic non-destructive in-situ measurements. The SWIR spectra have broad absorption peaks at ~ 1450 and ~ 1930 nm associated with H_2_O and the broad stair step band at ~ 2200 to 2360 nm corresponding to Si–OH vibrations, which is characteristic for opal or hydrated quartz (Fig. 3). However, absorption features that are characteristic for calcite are not observed in the hyperspectral SWIR images and spectra opposed to FTIR and XRD analyses, which were powdered prior to analysis and showed a mix of calcite and opal. Furthermore, SWIR spectra of white coatings of basaltic rock samples show similar spectra as the gypsum or opal spectra of the samples analysed in this study. FTIR spectra of the coralloid speleothems display mainly strong absorbance of Si–O bending vibrations at ~ 1100 cm^−1^, corresponding to opal, and of the υ2 at ~ 870 cm^−1^ and υ3 at ~ 1404 and ~ 1412 cm^−1^ CO_3_^2−^ bending vibrations corresponding to calcite (Fig. 5H, I). Small absorbance peaks at ~ 780 cm^−1^ in the speleothem samples correspond with Si–O stretching in the Si–O–Si groups. The split peak at ~ 1404 and ~ 1475 cm^−1^ and the small peak at ~ 871 cm^−1^ in Fig. 5I corresponds to the υ3 and υ2 vibrations of monohydrocalcite. Additionally, the intensity and shape of the absorbance peak corresponding to the O–H stretch at ~ 3500 cm^−1^ varies with each coralloid speleothem sample.

## Discussion

In this study, the secondary minerals are solely confined to the lava tubes, air pockets in shelly pahoehoe (rock cracks), which indicate that these secondary minerals formed in the subsurface as a result of diagenetic alteration of the overlying basaltic rock. Seepage waters percolate through the volcanic rock of tholeiitic composition (± 2.7 wt% total alkalis, Table 1), providing different ions into the solution from which the secondary minerals form. The formation of crystalline crusts made of gypsum and opal/calcite coralloid speleothems occurs in the lava tubes where water is observed on the walls, ceilings, cracks; or where water is dripping from lava stalactites. These secondary minerals, encompassing calcite, silica speleothems, gypsum, and thenardite, commonly originate as a consequence of seepage waters infiltrating the lava tube system, dissolving minerals in the process^6,16^. As these waters undergo transformations within the lava tube’s unique environmental conditions, such as shifts in pressure, temperature, or evaporation levels, they become supersaturated, prompting the minerals to precipitate and gradually shape the distinctive features found in the lava tubes^16^. However, the amount of crystalline crusts and coralloid speleothems in the HI-SEAS lava tubes is not formed to such a large extent as in some of the other lava tubes around the world^2,3,17–19^. The smaller abundance of these crystalline crusts and coralloid speleothems is likely dependent on the amount of rainwater that percolated through basaltic bedrock^20^. Although, the amount of secondary minerals is more extensive in the older lava tube (~ 1880 B.P.) since it formed a thicker soil layer with vegetation and, therefore, could have provided more leaching and transport of elements into the seepage water^3^. Consequently, the amount of secondary minerals is dependent on the concentration of ions provided by the host rock, soil formation, vegetation and meteoric water. Moreover, secondary mineral formation was limited on smooth cave floors as opposed to angular surfaces, such as in 'Āinakahiko Tube, except for the white powder deposits. A possible explanation is that airflow and seepage pathways play a role in speleothem formation as well since angular surfaces act as better nucleation sites rather than smooth surfaces. Furthermore, an increase of reactive surface could have been formed after collapsing and breaking of the lava tubes enhancing the formation processes.

The deposition of large quantities of white powders, in even very young lava tubes (A.D. 1983), indicates that secondary mineralization takes place rapidly after cooling. Similar sulphate deposits have been found in 2000 years old lava tubes in the COM basalts, Idaho, indicating that precipitation and preservation of these deposits in lava tubes occurs in post cooled lava tube conditions over a long period^5,21,22^. The sodium cation in the Na_2_SO_4_ complexes can derive from leaching of the seepage waters through the basaltic rock of which the solubility depends on the combination of temperature and relative humidity^6,18,21,23^. The sulphide anion can derive either from the breakdown of ferric iron sulphide rich minerals, as large amounts of hematite and goethite have been observed on Mauna Loa that formed from sulphide-rich rocks^24,25^, or from sulphuric acid provided by volcano gases^26^.

Mirabilite is only observed at two locations where the deposits displayed a crystalline salt-like morphology instead of white powder (Fig. 1B: SP2-B, KC4). Mirabilite is unstable at ambient temperatures and quickly loses its incorporated water by a lower humidity, transforming to thenardite^18^. This process is also observed in SWIR data of this study as the spectra are characteristic for mirabilite, while the samples were collected in the form of thenardite indicating that the samples transformed to its hydrous constituent prior to analysis. Therefore, relative humidity and temperature in the cave may play an important role in stabilizing mirabilite, preserving it from dehydration at the time of observation. Differentiation between the two minerals can also depend on the variation of moisture between wet and dry seasons^4,5^.

The crystalline gypsum crusts are generally associated with evaporation of seepage waters that infiltrate the lava tube (capillarity or drip), leaving behind crystalline patches on the cave walls^2,20,27^. The supply of the chemical compounds is likely similar to thenardite as the overhanging basalt is calcium-rich (± 10.3%; Table 1). In some patches, small puffballs are present (Fig. 4A), which could be related to gypsum balls that form typically in the deepest part of caves where there is a constant percolation of supersaturated water^27^. Studies show that the white coatings observed in the lava tubes are formed by air currents flowing through the lava tubes, which disperse and evaporate the seepage waters saturated in gypsum and silica leaving behind residual white coatings^10,20^. Consequently, these white coatings can act again as seeds for new secondary mineral formation^15,28^.

**Figure 6:**
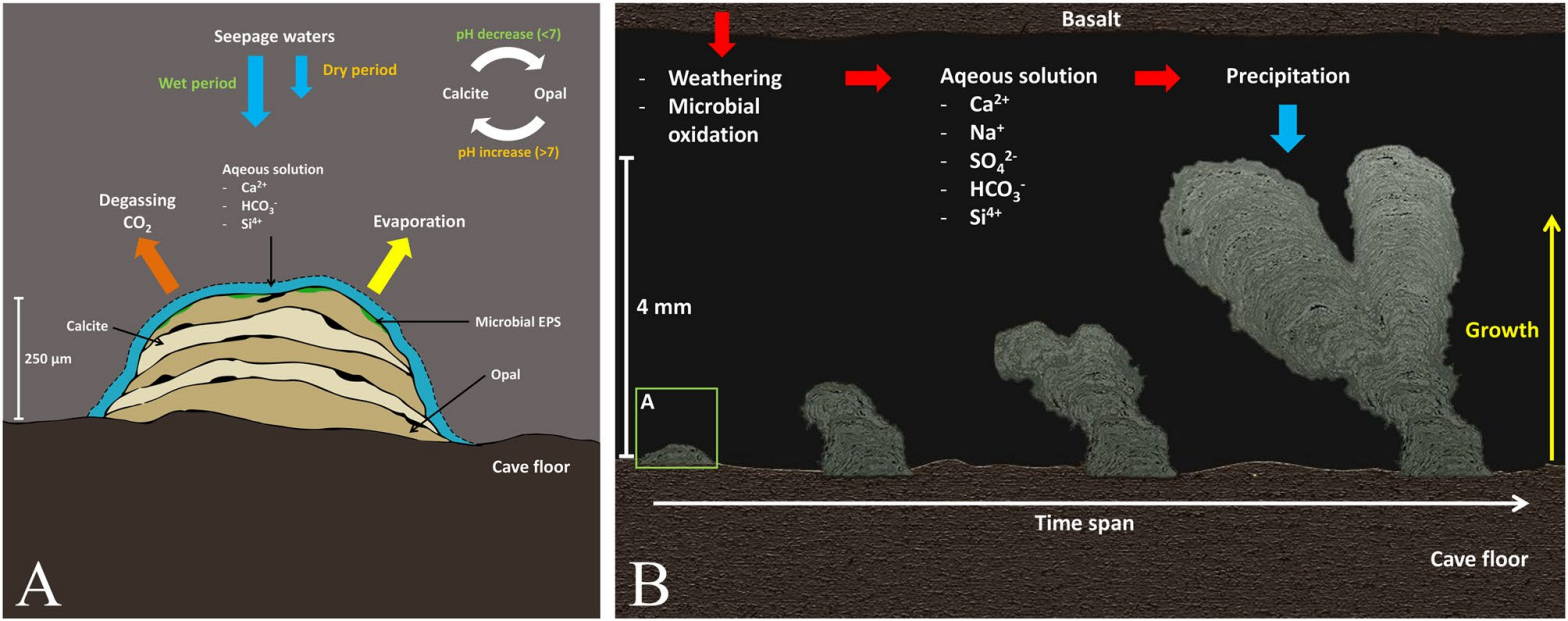
Schematic overview of secondary mineralization mechanics and coralloid speleothem formation. (**A**) Initial stage of speleothem formation related to amount of seepage water infiltration. (**B**) Basaltic weathering and precipitation of opal and calcite in coralloid speleothems over time.

SEM images show that the coralloid speleothems formed in alternating layers of opal-A and calcite, which indicates that the two different minerals, silica and Ca-carbonate, alternate in different phases (Fig. 5C, F). These types of opal speleothems can typically be found in lava tubes of which intermixed calcite and opal speleothems also have been reported^2,4,6^. The succession of these opal and calcite layers can be explained by repetitive growth due to changes in the pH of the water films that evaporates during alternating dry and wet periods as described in other studies (Fig. 6)^3,16,19,29^. It is likely that the coralloid speleothems formed after cooling of the lava tubes since lithified lichen is observed in 'Āinakahiko Tube, indicating that secondary mineral formation occurred simultaneously with lichen growth at this location. The coralloid speleothems have a small size even after, probably, existing for thousands of years (Fig. 5A). An explanation for the small size of the speleothems is that after the rapid formation of secondary minerals the growth rate of these speleothems wears down as the host rocks weather less over time, as is also observed in other lava tubes^6^. The relatively dry arid climate in the research area probably led to the preservation of the secondary minerals in their respective pristine environments^6,10^.

The presence of monohydrocalcite in samples of all lava tube systems is rare for such volcanic settings as it is unstable in natural conditions and transforms quickly to anhydrous carbonate minerals, such as calcite and aragonite^30^. However, it has previously been reported in a lava tube in Mexico^16^. The small peaks in the XRD patterns and the presence of water in FTIR spectra suggests that it is preserved in minor amounts within the calcite layers of the coralloid speleothems. The monohydrocalcite could have been formed and preserved in small quantities or it was substantially more present and is later altered to its stable constituent calcite^16^. The formation of monohydrocalcite can be attributed to several factors, such as evaporation of aerosols, biomineralization, slow formation kinetics, or crystallization from an Mg-rich precursor^30–34^. As for the presence of carbonate needles, these could be indicative of precursor aragonite that has altered to calcite as it is an unstable mineral (Fig. 5F, G). Aragonite can precipitate in speleothems at very low drip rates related to dry climate conditions or local hydrological variability^3,35^. It is noteworthy that calcite is not detected through SWIR spectroscopic analysis while it is detected by other analytical methods (FTIR, XRD) after being powdered. Either opal obscures the calcite absorption features within the short wave range or opal is the primary mineral and calcite formed as infillings. Therefore, it could be the case that SWIR spectroscopy detects the outer layer of the coralloid speleothems, which consists of opal.

There are some indications that microbial processes played a role in the formation of the coralloid speleothems in the research area. Firstly, the presence of nitrogen in a coralloid speleothem could indicate that nitrogen-fixing bacteria play a role in the genesis of these speleothems (Fig. 5E)^17,36,37^. The source of nitrogen is often ambiguous and could either derive inorganically from basaltic weathering in volcanic caves^2,38^, or organically from local vegetation, lichen, or animal feces. Nonetheless, nitrogen is only observed in the coralloid speleothems, suggesting that if these inorganic processes are responsible for the nitrogen supply, nitrogen should have been observed in other secondary minerals as well. Since vegetation and feces are scarce in the research area, nitrate could have probably been provided by the lichen present at the entrances of the lava tube systems. However, the coralloid speleothems are sampled from within the lava tubes, which has implications for the nitrogen source. If nitrogen is biotically mediated in the coralloid speleothems, it is probably fixated by lichen or diazotrophs. Secondly, the different branches of the coralloid speleothems formed on the lava tube ceilings, walls and floor show similarities to stromatolites and have relatively high porosity respective to the concentric layering of calcite and opal (Fig. 5A, C-F)^17,19,39^. Other studies found a variety of microbial communities in different mineralogical features in lava tubes on Mauna Loa that are typical for these types of oligotrophic environments^10,40^. In Fig. 5D rounded bacterial cells can be observed together with opal nanospheres in the vugs of the coralloid speleothems, which are often associated with microbial filaments and extracellular polymeric substances (EPS)^23,41^. These vugs in the coralloid speleothem can act as possible microenvironments for microbes affecting the alkalinity and pH of the seepage waters^19,39,42^. If similar crystalline crusts or coralloid speleothems are formed in lava tubes on Mars, microbes may be using the pores or crystals within the secondary minerals as microenvironments in which they can regulate the surroundings to their benefit. The potential availability of water and chemical gradients in lava tubes on Mars could provide an energy-rich environment, which microbes could have used to take advantage of different metabolic pathways^43,44^. Furthermore, this study shows that small pristine speleothems have been preserved in isolated parts of lava tube systems. Since hydrous alteration processes play a role on the Martian surface it is very likely that if similar deposits formed on Mars, they are conserved in similar isolated lava tubes that provide even more stable conditions than on Earth.

**Figure 7:**
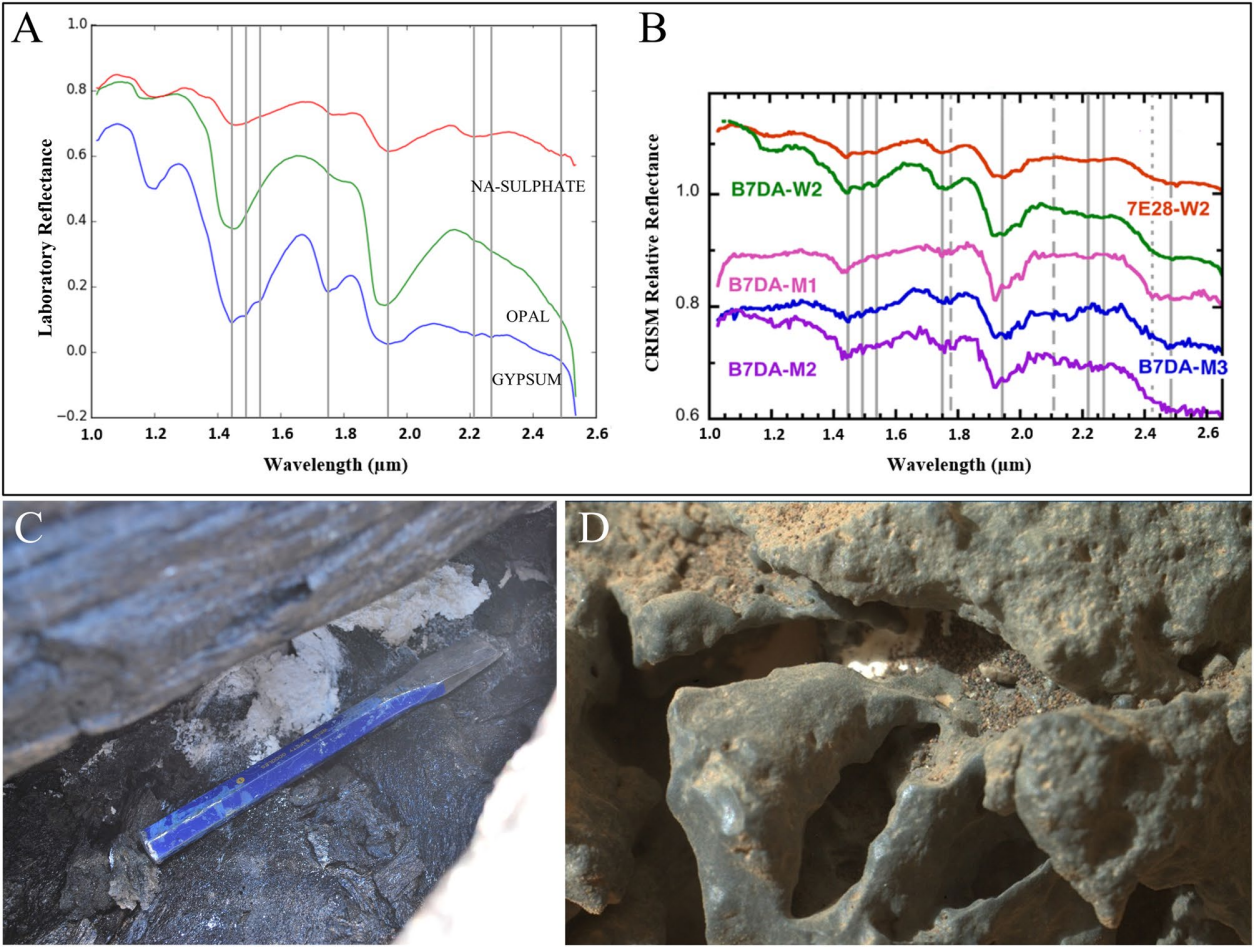
IR spectra of secondary minerals and images of white precipitates in basaltic environments. (**A**) Laboratory spectra of secondary minerals from this study. Grey lines represent different hydroxide absorption bands. (**B**) Spectra of LTD’s in the southern part of Noctis Labyrinthus near the Tharsis Bulge (modified from Weitz et al., 2013). These spectra are interpreted as gypsum-bearing and polyhydrated sulphate-bearing deposits. (**C**) Thenardite deposit in a cavity of shelly pahoehoe near the HI-SEAS habitat. (**D**) Image made by the Curiosity rover (on Sol 304) of a possible secondary mineral deposit in a rock crevice, Gusev Crater, although it could be CO_2_ or H_2_O ice deposits.

The occurrence of recent or past hydrological processes on Mars is indisputable, nowadays. Mars orbital missions have discovered a wide amount of light-toned deposits (LTD’s) on the Martian surface together with the presence of hydrous minerals including opal, carbonates, polyhydrated sulphates and gypsum bearing deposits, which are thought to be related to the movement of subsurface fluids^26,45–49^. The morphology and spectra of these Martian LTD’s show similarities to those observed in this study (Fig. 7). Opal-A and gypsum are identified in situ by Mars Exploration Rovers (MER) in Gusev Crater and Endeavor Crater of which the gypsum is thought to have been formed by dissolved calcium in seepage waters and sulphate that derived from aqueous oxidation of the surrounding rocks or was introduced by volcanic gas, comparable to the gypsum observed near HI-SEAS^25,26,50–52^. Furthermore, it is thought that opal on Mars already precipitates after minor aqueous alteration of silica-bearing rocks at low temperatures^47,53^. Martian carbonates are associated with olivine-rich and silica rocks similar to Hawaiian basalts, however, the amount is restricted to only a few basins^54–56^. The current lack of observed carbonates on the Martian surface could be explained by the either the morphology of the coralloid speleothems or the geographical location. This study showed that calcite and monohydrocalcite is only present within the coralloid speleothems together with opal, which could have obscured calcite as the dominant mineral in the short wave spectral range. Therefore, similar detection methods will not detect calcite in these types of secondary minerals. Regarding the geographical location of the secondary minerals, calcite is only observed deep inside lava tubes in the research area where it forms from seeping groundwaters. Therefore, remote detection of carbonates is likely hampered by the limited surface exposure of the carbonate minerals. Consequently, Ca-carbonates on Mars could be concealed from satellite spectrometers and solely be present in subsurface cavities, such as lava tubes. Furthermore, the rich CO_2_-rich atmosphere of Mars could have facilitated carbonate growth inside these lava tubes by means of mineral carbonation^57^. Na-sulphates are thought to be the most likely one to occur on the Martian surface from the polyhydrated sulfates as a result of evaporation^5,58,59^. Additionally, Martian surface rocks are more alkaline compared to the tholeiitic basalts of this study at some locations, such as Gale crater^60^, which has a higher potential for Na-sulphate deposition. Therefore, the analogous morphology of the white deposits in this study compared to those in Gale Crater could suggest a similar depositional environment of polyhydrated sulphates, since other sulphates (e.g., gypsum, bassanite, anhydrite) also have been observed in Gale Crater^61^.

## Summary and conclusion

The aim of this study was to assess the different types of secondary mineral deposits deep within lava tubes as analogue for Martian environments. SWIR spectroscopy was applied to mimic mineral spectroscopic detection methods used on Mars rovers and to acquire hyperspectral images of secondary minerals on basaltic surfaces. Secondary minerals were characterized as white powders (thernardite/mirabilite), crystalline crusts (gypsum) and coralloid speleothems (alternating opal and calcite layers). Our results show that monohydrocalcite and aragonite pseudomorphs are present in the samples, which is not common for these types of caves. EDS mapping and SEM images show the presence of nitrogen and microbial filaments in the coralloid speleothems, suggesting a microbial influence in the formation of these secondary minerals. Based on our observations and data, we conclude that if carbonates are embedded in a silicate matrix, such as opal, SWIR will not detect it in contrast to powdered samples analysed by FTIR and XRD, which can have implications for non-destructive analysis of these types of secondary minerals. Nonetheless, SWIR spectroscopy offers a supplementary instrument for field-based hyperspectral mapping of (hydrous) secondary minerals in lava tubes. The origin of the secondary minerals in lava tubes near HI-SEAS together with potential evidence of microbial activity contributes to our understanding of subsurface processes on other terrestrial bodies such as Mars and our search for traces of extraterrestrial life. Further research is needed on secondary minerals in lava tubes near HI-SEAS in order to understand the origin of nitrogen in the coralloid speleothems and the precise formation mechanisms of the secondary minerals.

## Material and methods

### Field work

During a two-week lunar simulation, the human limitations were tested regarding scientific field research of secondary mineralization and lava tube exploration. Field research was conducted in specialized analogue astronaut suits together with drone operations. 28 samples were collected from the three large parallel lava tube systems with different formation ages (Fig. 1B). The sampling took place within the lava tubes to mimic subsurface oligotrophic conditions similar to Mars and was done with great care for the surroundings to preserve the pristine environment of the lava tubes. Basaltic rock samples with secondary mineral deposits on the surface were sampled for Short Wave Infrared Spectroscopy (SWIR) after which these deposits were scraped from the basaltic rock surface for the other analytical techniques. The samples were enclosed in aluminium foil and only touched by gloved hands for preservation and to prevent excessive contamination of the samples.

### Sampling site description

Two of the three lava tube systems are named after the lava flows described by Trusdell and Lockwood (2017): *'*Āinakahiko Tube and 'Āinahou Tube (Fig. 1). The lava tube that is located in the youngest lava flow near HI-SEAS is referred to as Columbus Tube as this lava flow is not yet named in previous studies. The bedrock of the oldest lava tube, *'*Āinakahiko Tube, is strongly weathered to brown and reddish within the vicinity of locally small bushes growing on the surface. Two segments of this lava tube were accessible at three locations and resides 2–2.5 m in the subsurface (Fig. 1B, AT2). The most northern segment was accessible from both sides whereas the middle segment was accessible by one entrance (Fig. 1B, AT4). The lava tube has ~ 9 skylights of which most are collapsed. The accessible lava tube entrances are characterized by the growth of small white to light green stalactites along with some moss patches on the ceiling and walls (Supplementary Fig. S2A). Additionally, small quantities of fine-grained white powder are accumulated on the floor or in cracks. Inside the lava tube system, the walls and ceilings are covered in a white faint shallow coating on the walls together with the small stalactites (1–3 mm.) and more developed coralloid speleothems (7–15 mm). A white to green crystalline botryoidal crust is locally present as small patches on the walls. In an isolated area of the lava tube with only one entrance (2.5 m. wide and 1 m. height) the coralloid structures are exceptionally well developed forming extensively in nearly every possible location of the cave, such as solidified open gas bubbles or even loose rocks on the ground (Fig. 5A). Although coralloid speleothems are formed on the floor, there is no obvious correlation with the coralloid speleothems on the ceiling. Striking is that the coralloid speleothems are the main observed feature in this part of the lava tube system with locally only botryoidal crystalline crust patches.

The 'Āinahou Tube is a large lava tube system in which the number of skylights near the HI-SEAS stretch up to a distance of at least 1800 m. The majority of the secondary minerals were sampled from this lava tube system, which consists of three large accessible segments and some skylights in the north (Fig. 1B). This lava tube system is younger than the *'*Āinakahiko Tube and, therefore, its surface and subsurface (light grey to brown) is less affected by hydrological processes together with little plant growth. The segments of the lava tube system are located ~ 3 m under the subsurface varying from 1.5 m to 6 m in height (Supplementary Fig. S2C). The entrances are mainly characterized by a significant amount of green and yellow mosses, white mineral coatings, white powder deposits, and large patches of white to green crystalline botryoidal crusts with locally orange corrosion on the walls, ceiling, and in the cracks. The skylights of the lava tube system are associated with loose rock debris on the floor together with mosses on the walls to the extent of where the sunlight reaches during the day, whereas the dark parts of the lava tube are defined by smooth dark brown walls and flat floors with locally lava stalactites on the ceilings (Supplementary Fig. S2D). The lava stalactites are excellent speleothems for secondary minerals to form on their surface at these locations. These lava stalactites are mainly covered by white coating and white green to yellow small botryoidal and coralloid speleothems. The ambient air in the lava tube segments has relatively high humidity, locally water has been observed dripping from some lava stalactites (Supplementary Fig. S2E). The northern segment is more humid than the southern segment as the floor is slippery and very wet and has a significant amount of secondary mineralization in this part of the lava tube system. The middle segment of the 'Āinahou Tube (Figs. 1B: SP2) can be accessed by an entrance in the north and in the south (Fig. 1B) and is relatively isolated from the external environment with secondary mineralization confined to white coatings on the walls and floor.

Columbus Tube is the longest and youngest lava tube system compared to the other lava tube systems and, therefore, the bedrock of the lava flow is only slightly weathered, maintaining its black oily lustre. The Columbus Tube is observed in the field as several collapsed elongated skylights, 3 to 20 m. deep, that split in two directions towards the north (Fig. 1B). Some of the skylights are surrounded by pahoehoe flows, which are characterized by a silver lustre, that seem to come from the skylights draping over the underlying lava flows. The ceiling of this lava tube contains lava stalactites covered by the white coating and small botryoidal speleothems similar to those observed in parts of 'Āinahou Tube. During this study the skylights of Columbus Tube were solely accessible by drone with exception of sample location CT7, where a considerable amount of fine-grained white powder was observed on the lava tube floor together with minor abundances of small coralloid speleothems on the walls.

### X-ray fluorescence (XRF)

To compare the composition of the secondary minerals with the composition of the basaltic host rock from which these secondary minerals formed, powdered bulk samples of several basaltic rocks have been analysed by wavelength dispersive XRF using a Panalytical AxiosMax for major elemental concentrations. These powder samples were preheated in an oven at 1000 °C for ~ 1 h to dry the samples for loss of ignition (LOI). After drying of the samples, 6 g of flux was added to 1 g of the powder sample, which was afterwards mixed, extensively. The mixed powders were emptied into platinum crucibles to forge 40 mm fused glass beads in platinum casting dishes by heating the samples in a Panalytical Eagon2. The samples are calibrated to four Vrije Unversiteit (VU), Amsterdam, in-house standards to ensure the sum of concentrations of these standards stays above 99%. It has to be noted that heating of the samples in the oven causes an oxidation reaction in which all the Fe^2+^ oxidises to the heavier Fe^3+^. Therefore, the oxidation state of the Fe was ignored and expressed as total ferrous iron (FeO).

### Fourier transform infrared (FTIR) spectroscopy

Attenuated Total Reflectance (ATR) FTIR spectroscopy was used for mineral and chemical compound characterization of the secondary minerals by using a Bruker Vertex70 FTIR spectrometer at the faculty of Geo-Information Science and Earth Observation (ITC) of the University of Twente (The Netherlands). Twelve samples were grounded into fine homogenized powders with a corundum mortar and pestle and heated at 85 °C for two hours to dry out and to prevent evaporation of extra moisture in the samples during measurements that can affect the intensity of the peaks in the spectra corresponding to H_2_O. Spectra are measured in absorbance with a resolution of 4 cm^−1^ between 600 and 4000 cm^−1^ with a total of 16 scans. Samples were analysed in duplicates to validate the precision of the instrument after which baseline correction was applied to every recorded spectrum. After each measurement, the sample’s surface, as well as the Platinum-ATR accessory with diamond crystal, were cleaned with ethanol to prevent cross-contamination. Spectra are made in ENVI Spectral Library format with the use of Hyperspectral Python 3 (HypPy3) software^62^. Absorption peaks of the FTIR spectra of the samples are manually interpreted by using the online spectral library RRUFF^63^ and existing literature (Supplementary Table S2).

### Short wave infrared (SWIR) imaging spectroscopy

Hyperspectral images were acquired for 15 samples of which secondary minerals as well as the basaltic host rocks by using a Specim SWIR-LVDS-N25E camera and OLES30 lens from 1000 to 2500 nm in 288 bands at 256 µm pixel size mounted in a laboratory stage setup (Specim, Spectral Imaging Ltd, Oulu, Finland). The samples were placed in a box containing quartz sand and horizontally levelled for image acquisition after which it was scanned under dark laboratory conditions for high spectral resolution image files. Secondary minerals were left on the basaltic rock surface on which it formed in order to compare spectra of the secondary minerals with those of the host rock. Generally, the amount of reflectance is related to the grain size of the mineralogy and the amount of absorption by hydroxyl overtones, whereas the change in reflectance with wavelength is related to mineral composition^64,65^. The data is processed by HypPy3 and ENVI software, which reduced noise without altering the spatial and spectral features in order to improve spectral image quality^66,67^. Albedo images are taken from each sample together with several wavelength maps, which show the wavelength positions of the deepest absorption features between specific wavelength ranges in colour images^67^. Additionally, every pixel in the albedo images provides its own characteristic spectrum. The absorption features and general shape of individual SWIR spectra are manually interpreted by using the online spectral library USGS Spectral Library Version 7^68^ and existing literature (Supplementary Table S2).

### Scanning electron microscopy (SEM) and energy-dispersive X-ray spectroscopy (EDS)

The material of nine handpicked secondary mineral samples was cast in epoxy resin and sputter-coated with a carbon film before being examined through SEM analysis to determine elemental compositions and microstructures. The backscattered electron (BSE) and energy-dispersive X-ray spectroscopy (EDS) mode of a JEOL JCM 6000 Plus (VU, Amsterdam) was used for elemental mapping and spot analysis of the samples. In the BSE mode surface, images were obtained from the secondary minerals in the epoxy resin with a beam current of 100 mA and beam voltage of 15 kV.

### X-ray diffraction (XRD)

Heterogeneous powdered samples were made of eight secondary mineral samples for mineral identification to verify the IR spectroscopy patterns using XRD at Cardiff University (UK). Sub-samples were used from these powder samples to obtain X-ray powder diffraction patterns by using a Philips PW-1710 Automated Powder Diffractometer with monochromated Cu Kα radiation at 35 kV 40 mA. The Identification of the diffractograms was manually performed by using Match! 3 software in which the peaks were referenced to the ICDD database. https://www.crystalimpact.de/match.

### Supplementary Information

Supplementary Information.

## Data Availability

The datasets generated during the current study are available in the Mendeley Data repository (https://data.mendeley.com). 10.17632/37yg6zgmh9.1.
