# Supplementary material for: Multitechnique characterization of secondary minerals near HI-SEAS, Hawaii, as Martian subsurface analogues
Source: Sci Rep. 2023 Dec 18;13:22603. doi: 10.1038/s41598-023-48923-7 (PMC10730813; doi:10.1038/s41598-023-48923-7)
Supplement: Supplementary file 1 — Supplementary Information. [file 41598_2023_48923_MOESM1_ESM.docx]

Supplementary information – Scientific Reports

**Multitechnique Characterization of Secondary Minerals near HI-SEAS, Hawaii, as Martian Subsurface Analogues**

Sebastian Mulder^1,2*^, Frank van Ruitenbeek^3^, Bernard Foing^4^ and Mónica Sánchez-Román^2^

*^1^Energy and Sustainability Research Institute Groningen, Faculty of Science and Engineering, University of Groningen, Nijenborgh 6, 9746AG Groningen, the Netherlands*

*^2^Earth Sciences Department, Sciences Faculty, Vrije Universiteit Amsterdam, de Boelelaan 1085, 1081HV Amsterdam, the Netherlands*

*^3^ Department of Applied Earth Sciences, Faculty of Geo-Information Science and Earth Observation, University of Twente, Drienerlolaan 5, 7500AE Enschede, the Netherlands*

*^4^LUNEX/ILEWG EuroMoonMars & Leiden Observatory, Universiteit Leiden. Niels Bohrweg 2, 2333 CALeiden, the Netherlands*

Corresponding author: sebastian.mulder@rug.nl

| **Table S1**  Morphology and mineral composition of the secondary minerals. | | | | |
| --- | --- | --- | --- | --- |
| **Samples** | **Morphology** | **Mineralogy** | **Composition** | **Cave system** |
| SM-B | White powder | Thenardite, Mirabilite | Na_2_SO_4_(·10H_2_O) | Shelly pahoehoe |
| KT1-C | Crystalline crust | Gypsum | CaSO_4_·2H_2_O | 'Āinahou |
| KT2-B | Coralloid/ botryoidal | Opal-A, (Monohydro)calcite | SiO_2_·nH_2_O, CaCO₃, CaCO₃·H_2_O | 'Āinahou |
| KC2-A | White powder | Thenardite | Na_2_SO_4_ | 'Āinahou |
| KC2-B | Coralloid/ botryoidal | Opal-A, Calcite, Gypsum | SiO_2_·nH_2_O, CaCO₃, CaSO_4_·2H_2_O | 'Āinahou |
| KC3-B | White powder | Thenardite | Na_2_SO_4_ | 'Āinahou |
| KC4-B | White powder/ salt | Thenardite, Mirabilite | Na_2_SO_4_(·10H_2_O) | 'Āinahou |
| SP2-B | White powder/ salt | Thenardite, Mirabilite | Na_2_SO_4_(·10H_2_O) | 'Āinahou |
| AT2-A | Crystalline crust | Gypsum | CaSO_4_·2H_2_O | *'*Āinakahiko |
| AT2-B | Coralloid/ crystalline crust | Opal-A, Calcite, Gypsum | SiO_2_·nH_2_O, CaCO₃, CaSO_4_·2H_2_O | *'*Āinakahiko |
| AT4-B | Crystalline crust/ globules | Gypsum | CaSO_4_·2H_2_O | *'*Āinakahiko |
| AT4-D | Coralloid | Opal-A, (Monohydro)calcite | SiO_2_·nH_2_O, CaCO₃, CaCO₃·H_2_O | *'*Āinakahiko |
| CT7-A | White powder | Thenardite | Na_2_SO_4_ | Columbus (entrance) |
| CT7-C | Coralloid/ thin stalactites | Opal-A, (Monohydro)calcite, Gypsum | SiO_2_·nH_2_O, CaCO₃, CaCO₃·H_2_O, CaSO_4_·2H_2_O | Columbus (entrance) |

Figure S1 : SWIR wavelengths maps of samples AT4-B (gypsum) and AT2-B (gypsum and opal) on basaltic rocks at different wavelengths distinguishing the gypsum from the opal in sample AT2-B.


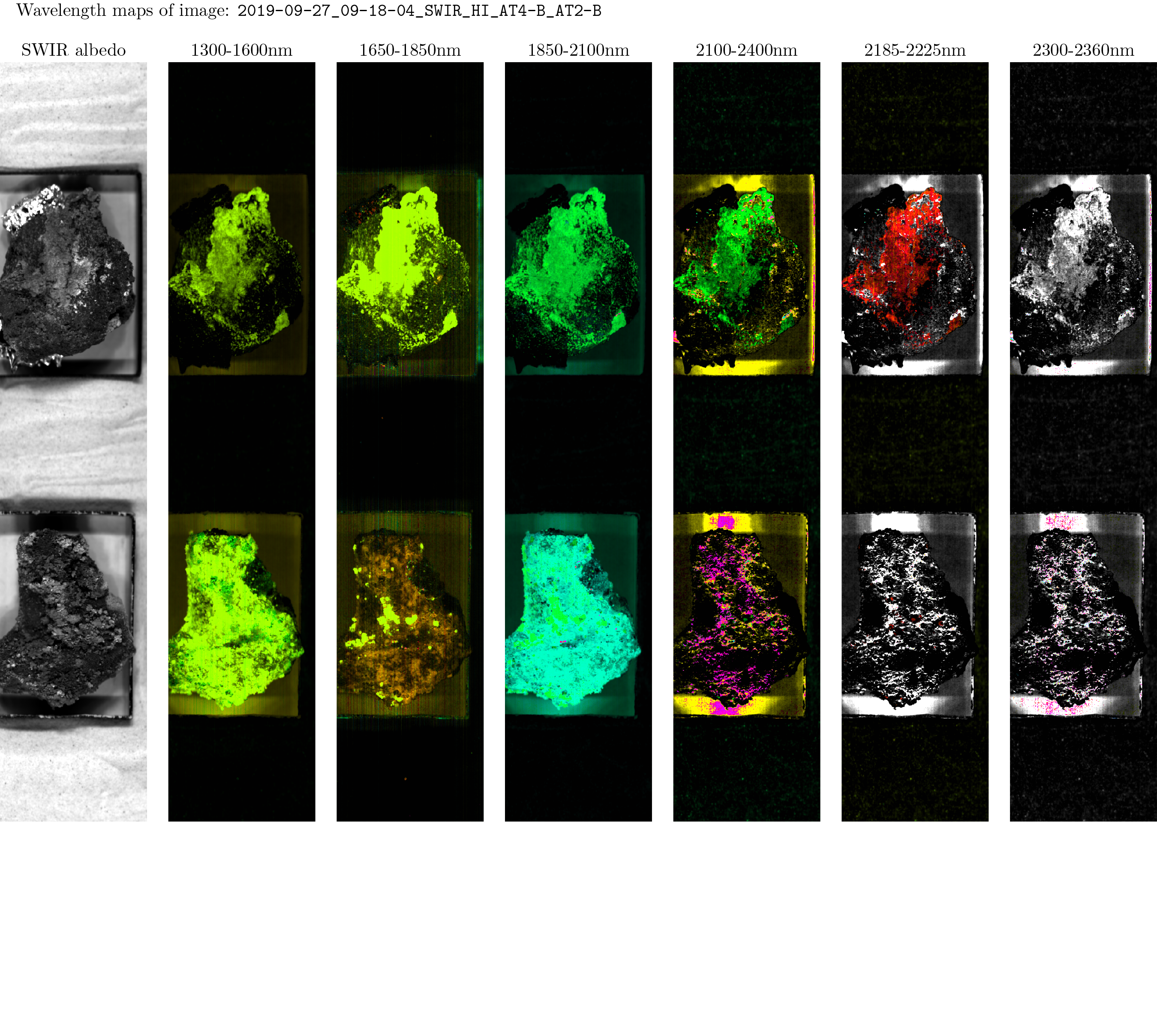

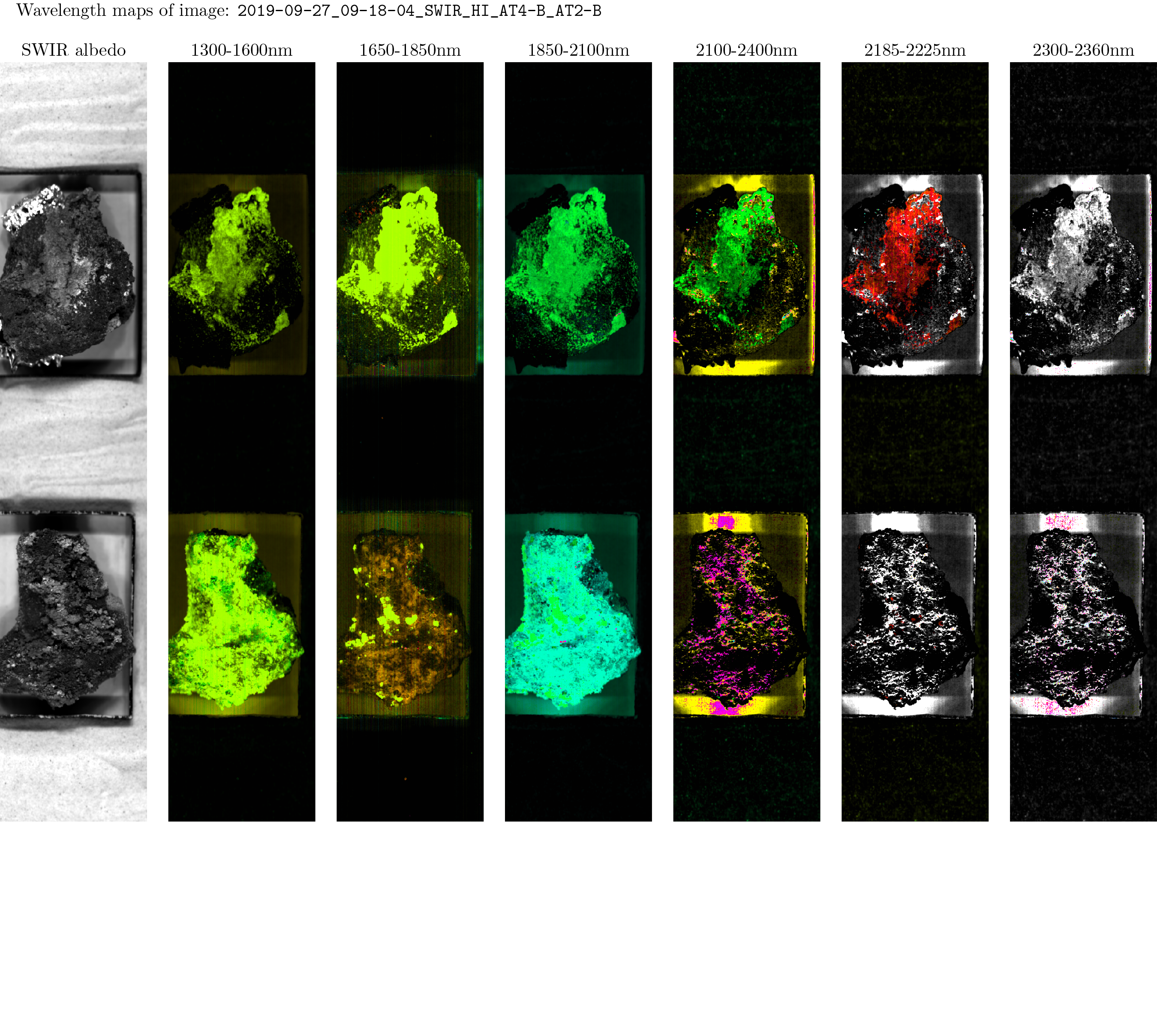

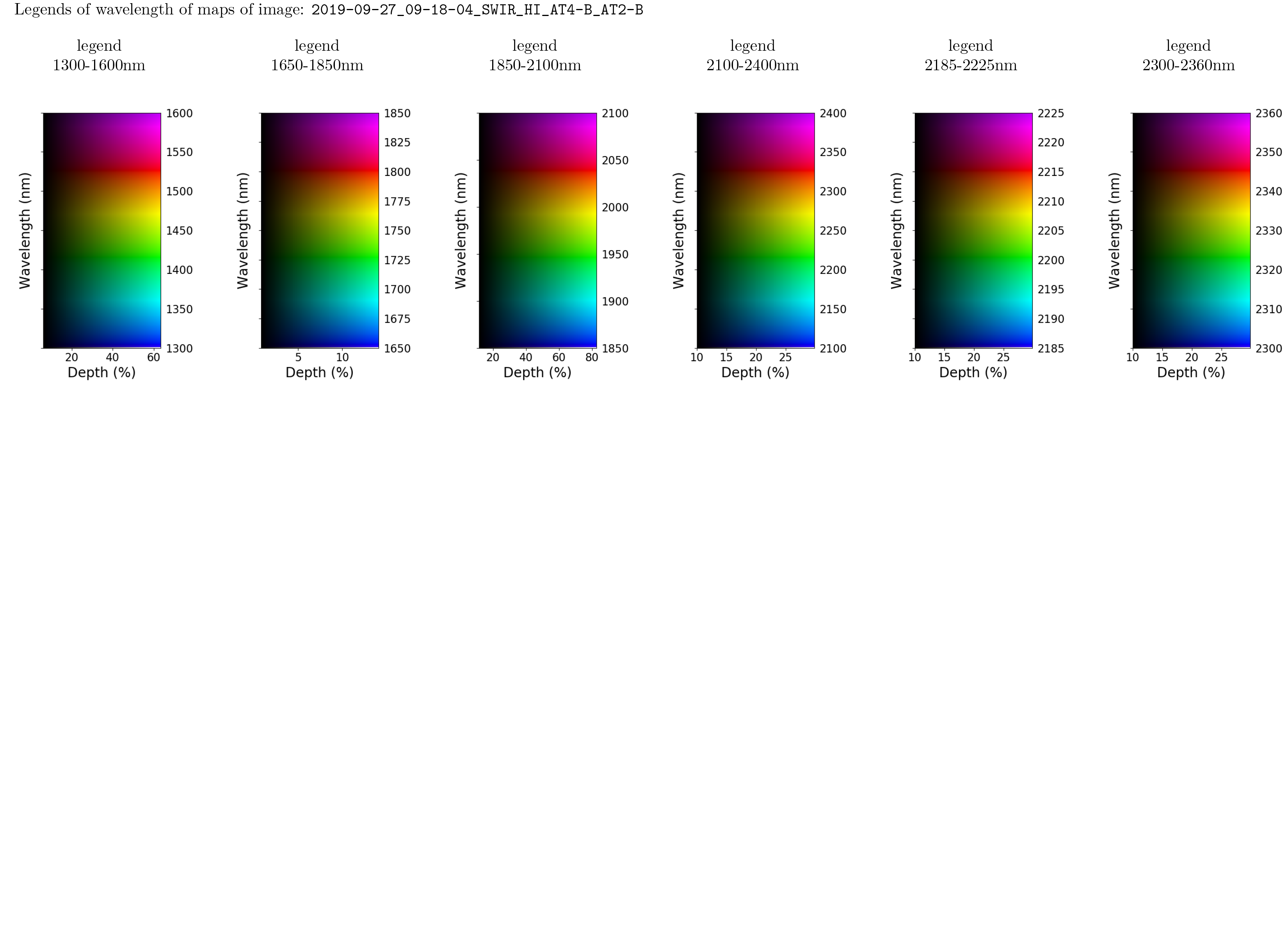


AT2-B


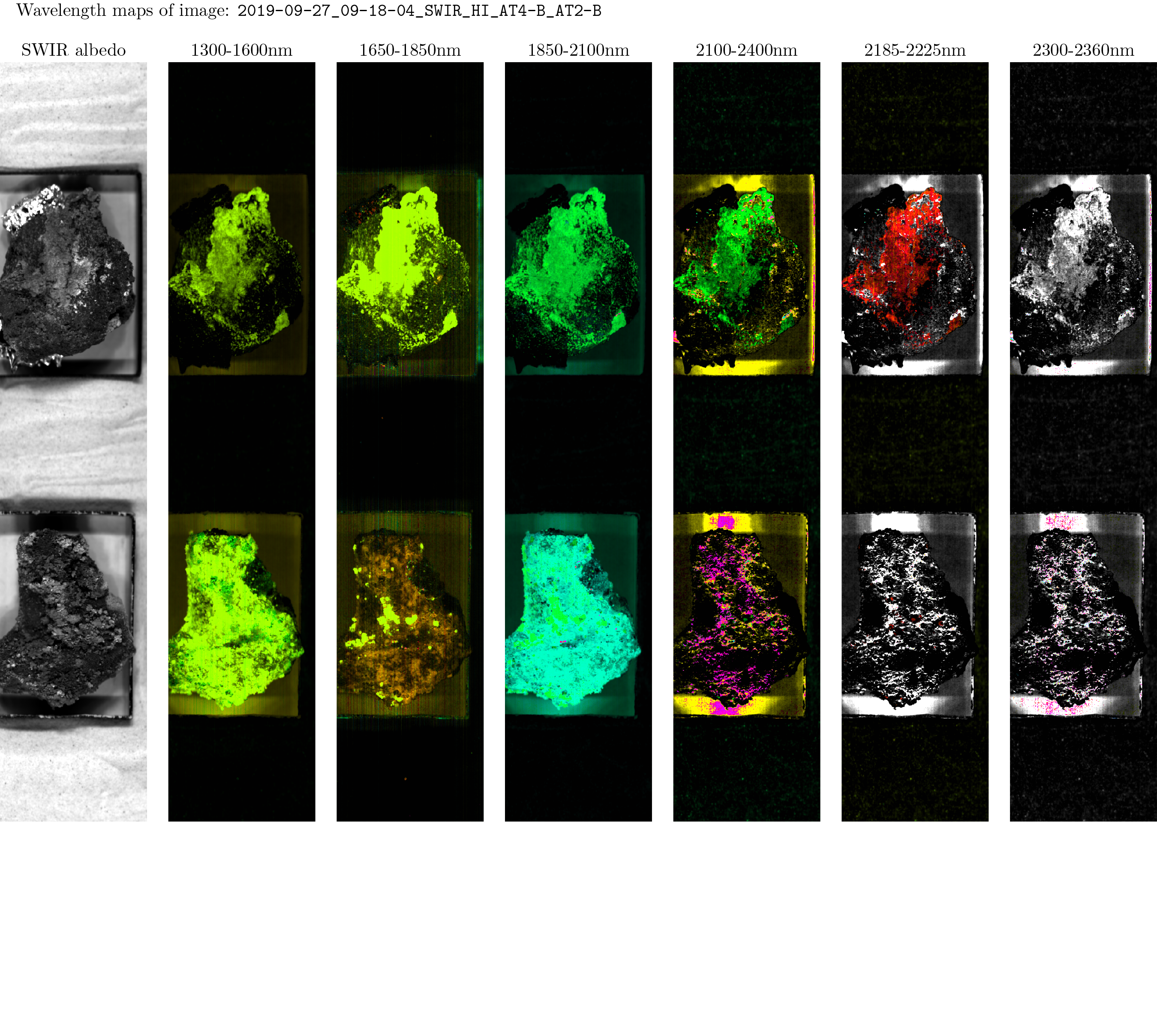


AT4-B


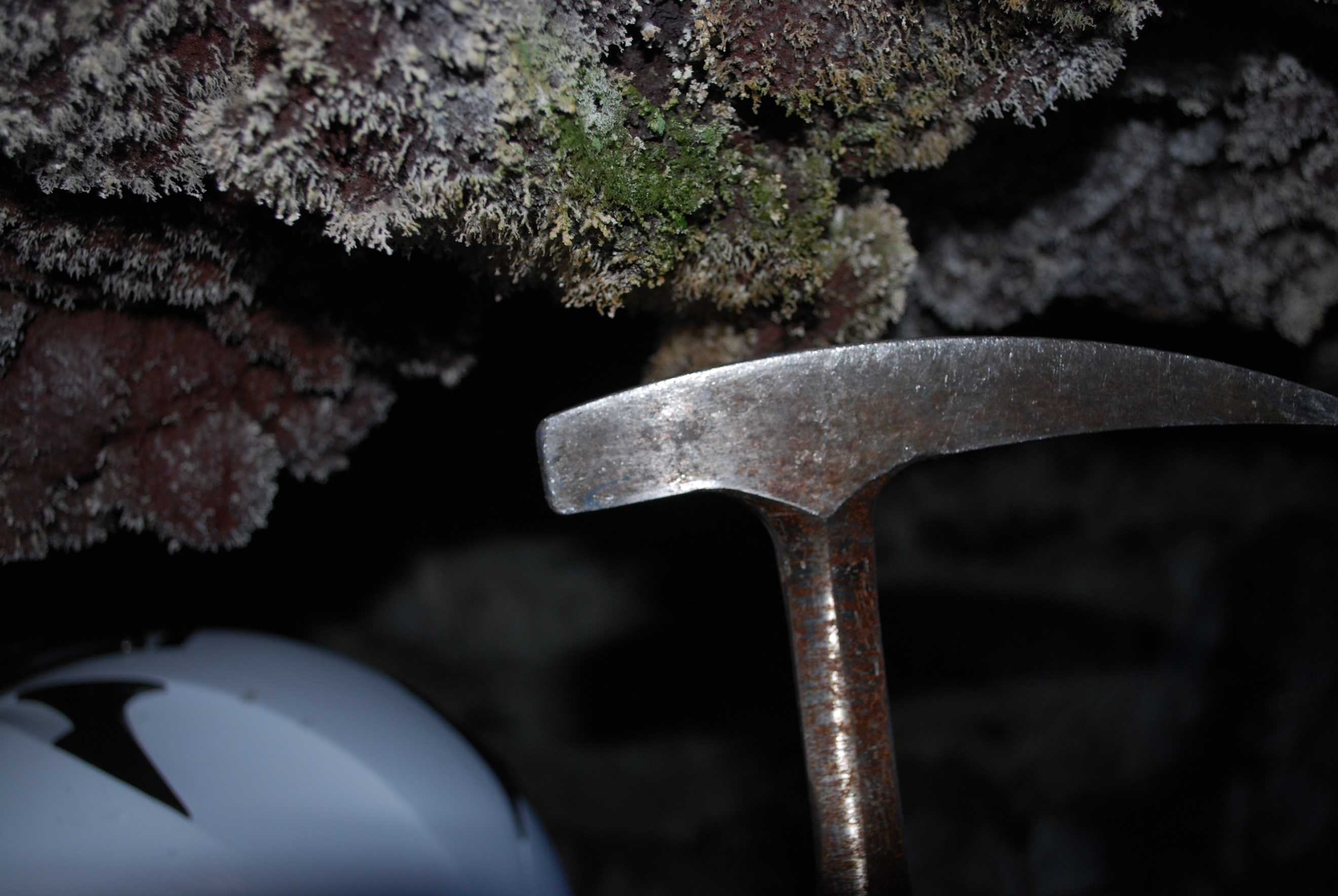


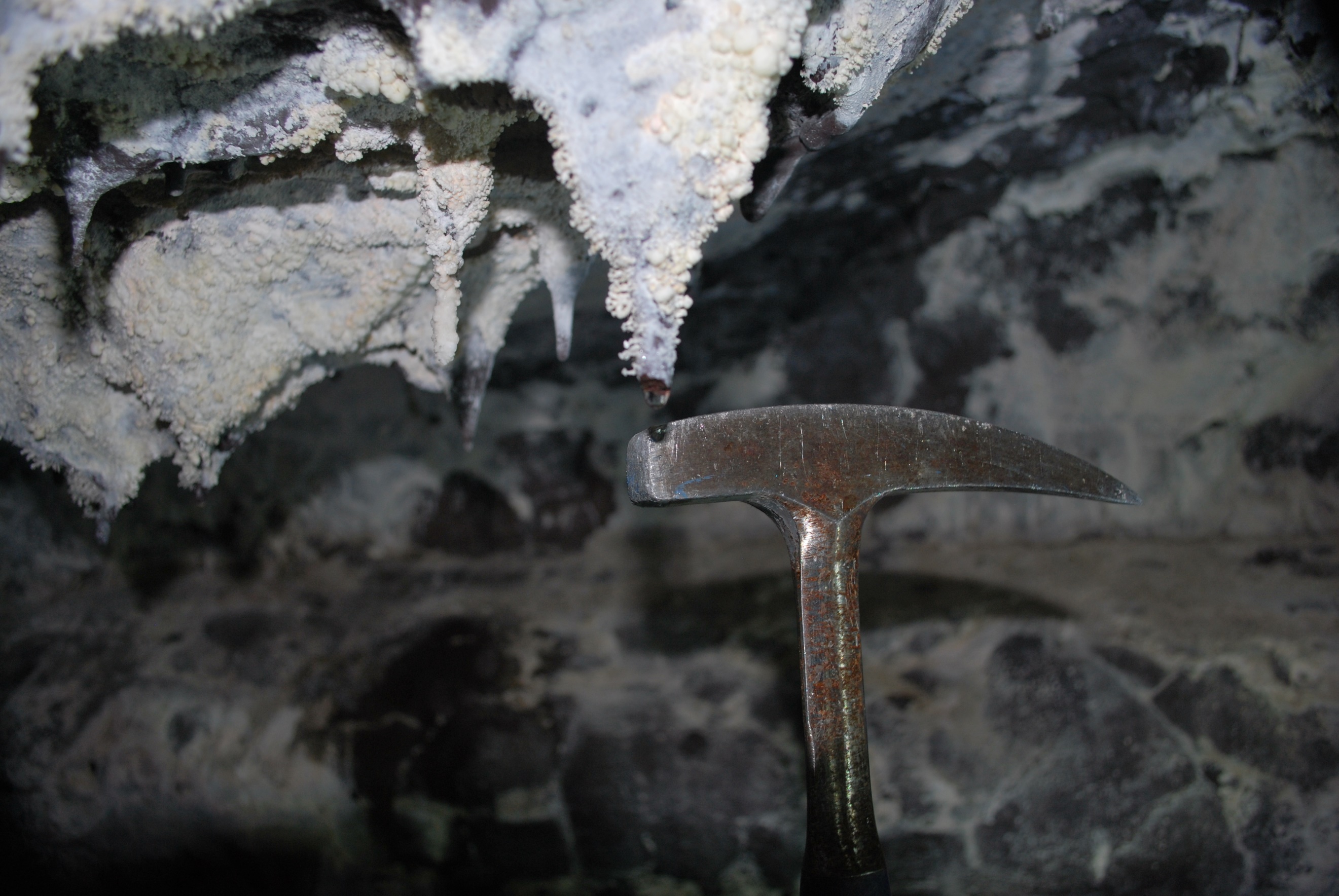

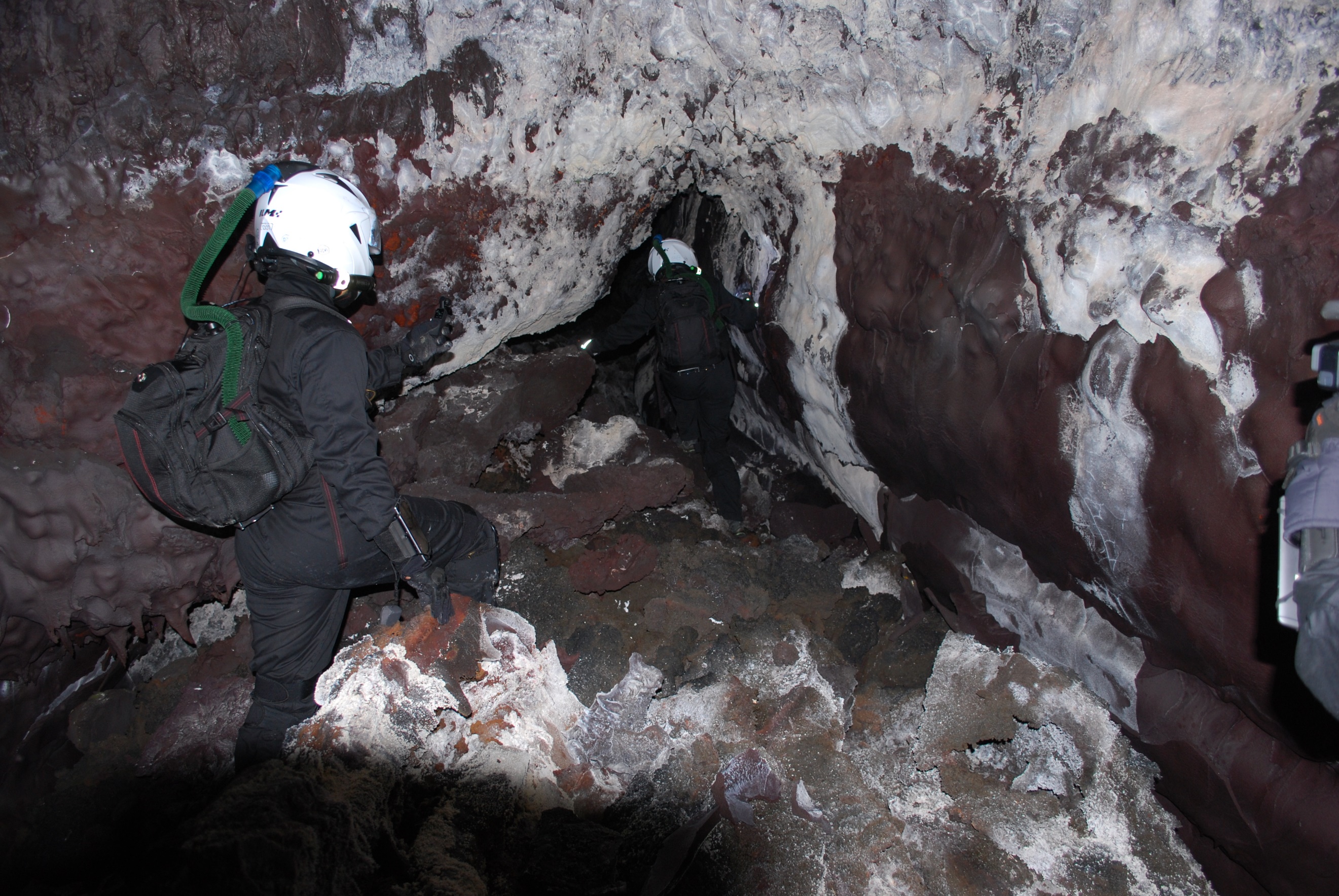

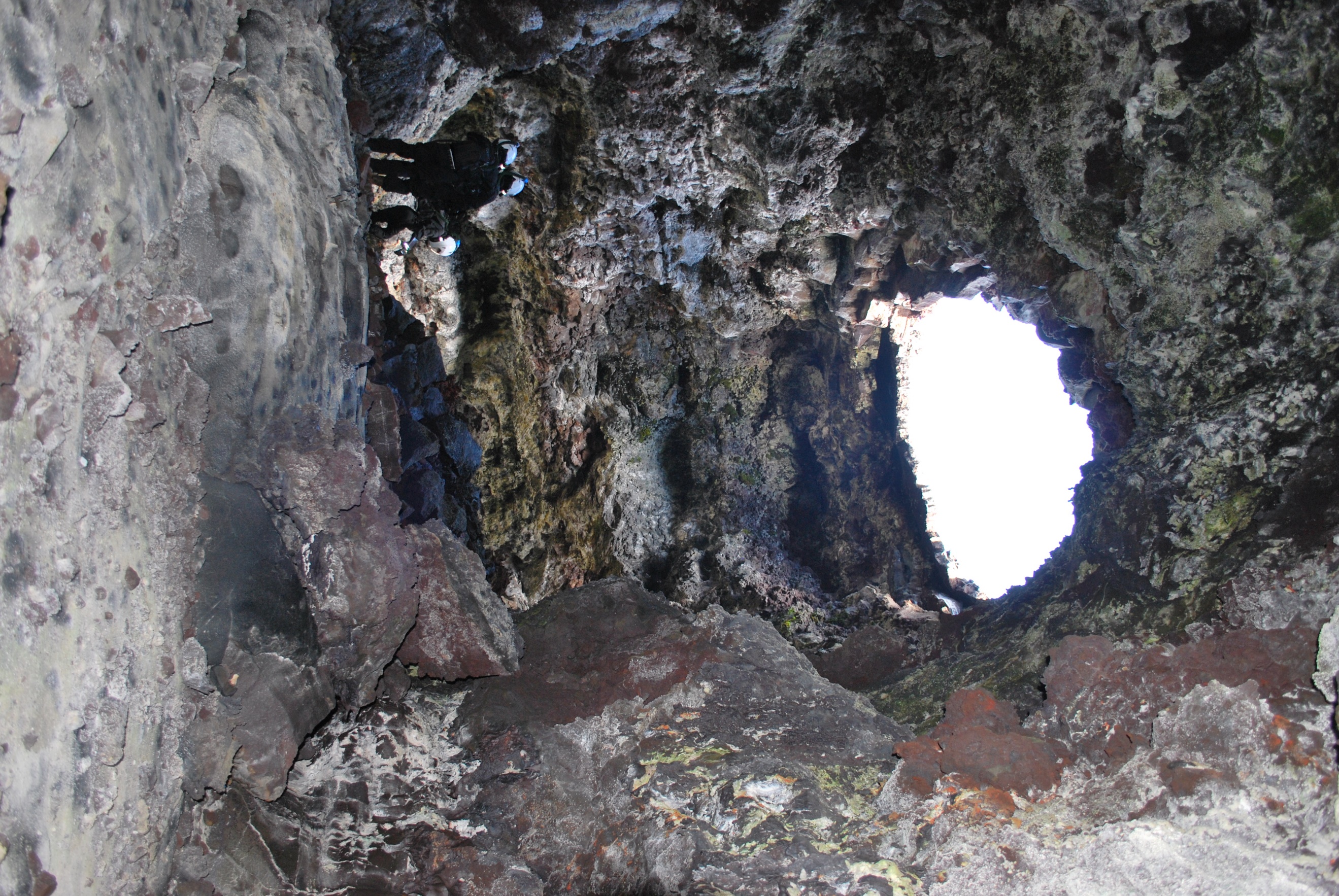

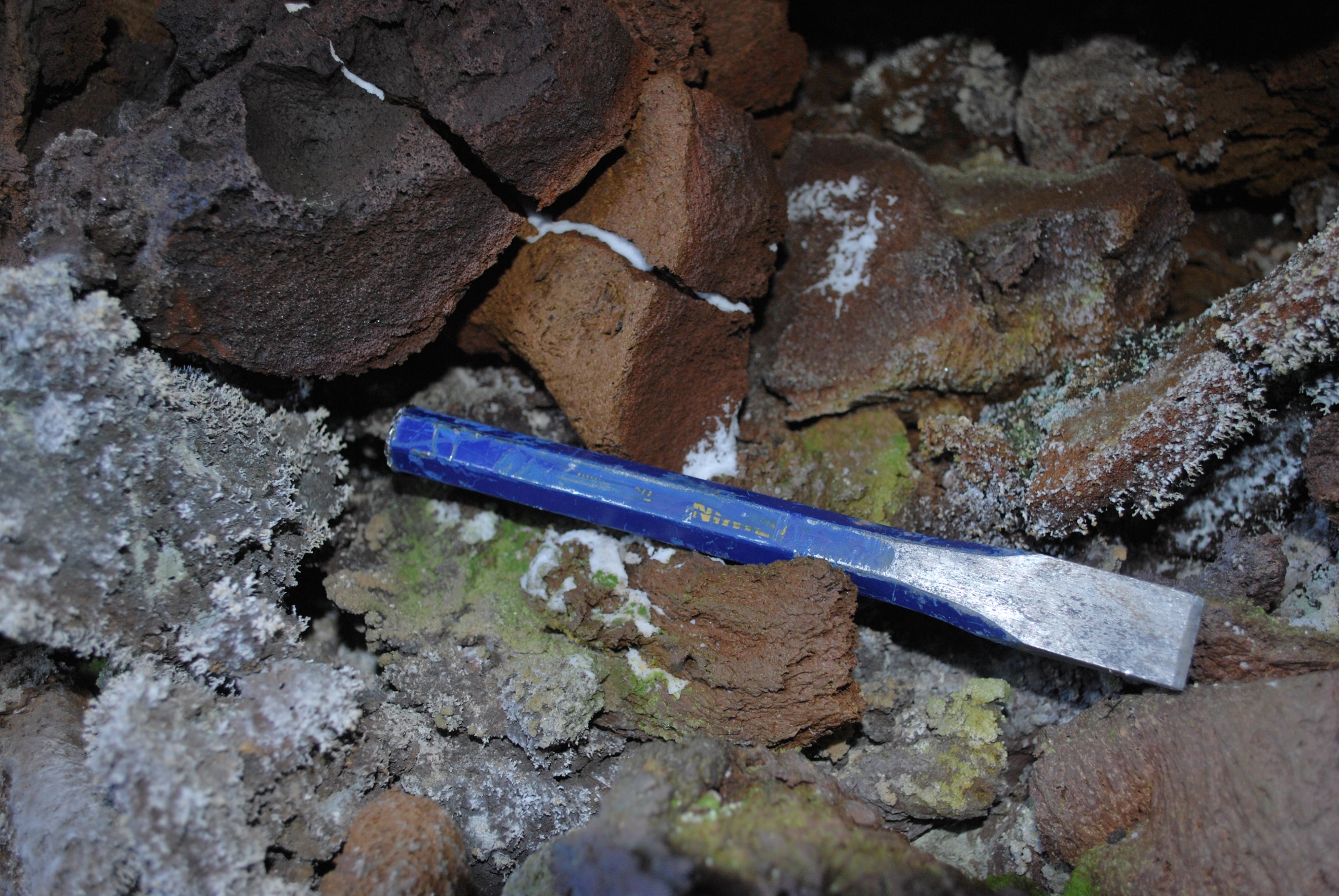


A

B

C

D

E

WP

CC

CS

Figure S2: Characteristics of three lava tubes systems with the respective secondary mineral deposits. A) A variety of secondary mineral deposits at a lava tube entrance of 'Āinakahiko Tube (AT4). WP= sulphate powder (Thenardite), CC= crystalline crust (Gypsum), CS= coralloid speleothem (Opal and Calcite). B) Lithified lichen on the ceiling next to dark green active lichen at lava tube entrance AT2. Size of the image is approximately 10 cm wide. C) One of the many skylights in the area, which reach deep into the subsurface. A wide abundance of secondary minerals covering the lava tube surfaces can be observed. D) Lava tube segment (KT2) with smooth surfaces and lava stalactites. E) Coralloid speleothems on lava stalactites at sample location KT2. Water dripping from a lava stalactite (yellow arrow).

| **Table S2**  Literature used in this study regarding IR spectroscopy of secondary mineral deposits. The references are stated at the bottom of the Supplementary Information file. | | |
| --- | --- | --- |
| **Mineral** | **Analytical Method** | **Reference** |
| Na-sulphates | FTIR | Coates, 2000; Richardson et al., 2012 |
|  | SWIR | Howari et al., 2000b; Kratt et al., 2006; Farifteh et al., 2008; Mayer et al., 2009 |
| Gypsum | FTIR | Backwell et al., 2018; Coates, 2000 |
|  | SWIR | Clark et al., 1990; Sun and Miliken, 2020 |
| Opal/hydrated quartz | FTIR | Coates, 2000; Miller et al., 2014; Vogel et al., 2016 |
|  | SWIR | Hauff et al., 2008; Smith et al., 2013; Canet et al., 2015; Calvin and Pace, 2016; Sun and Miliken, 2020 |
| Calcite | FTIR | Müller et al., 2014; Preston et al., 2014; Vogel et al., 2016 |
|  | SWIR | Clark et al., 1990; Sun and Miliken, 2020 |
| Monohydrocalcite | FTIR | Al Omari et al., 2016 |

ZAF Method Standardless Quantitative Analysis(Oxide)

Fitting Coefficient : 0.1116

Total Oxide : 24.0

Element (keV) Mass% Sigma Mol% Compound Mass% Cation K

C K 0.277 36.91 0.12 56.33 C 36.91 0.00 17.1474

Ca K 3.690 6.79 0.06 3.11 CaO 9.50 3.54 28.1836

Mg K 1.253 0.18 0.02 0.14 MgO 0.30 0.16 0.3288

N K 0.392 24.13 0.37 31.57 N 24.13 0.00 13.9976

O 18.39

S K 2.307 0.20 0.02 0.11 SO3 0.49 0.13 0.6387

Si K 1.739 13.40 0.12 8.75 SiO2 28.67 9.96 39.7039

Total 100.00 100.00 100.00 13.78

Acquisition Parameter

Instrument : JCM-6000

Acc. Voltage : 15.0 kV

Probe Current: 1.00000 nA

PHA mode : T3

Real Time : 32.38 sec

Live Time : 30.00 sec

Dead Time : 7 %

Counting Rate: 7390 cps

Energy Range : 0 - 20 keV

Figure S3 : Quantitative analysis of coralloid speleothem sample KC2-B.

References Supplementary Information S2

1. Coates, J., 2000. Interpretation of infrared spectra, a practical approach. *Encyclopedia of analytical chemistry: applications, theory and instrumentation*.
2. Howari, F., M., Goodel, P., Mizymoto, S. and Penn, B., 2000. Spectral properties of gypsum, halite and their mixtures: Fourteenth International Conference and Workshop on Applied Geologic Remote Sensing (An abstract), Las Vegas. *Nevada, Nov*, pp.6-8.
3. Kratt, C., Coolbaugh, M. and Calvin, W., 2006. Remote detection of Quaternary borate deposits with ASTER satellite imagery as a geothermal exploration tool. *Geothermal Resources Council Transactions*, *30*, pp.435-439.
4. Farifteh, J., Van der Meer, F., Van der Meijde, M. and Atzberger, C., 2008. Spectral characteristics of salt-affected soils: A laboratory experiment. *Geoderma*, *145*(3-4), pp.196-206.
5. Mayer, D. P., Arvidson, R. E., Wang, A., Sobron, P., & Zheng, M. P., 2009. Mapping minerals at a potential Mars analog site on the Tibetan Plateau. In *Lunar and Planetary Science Conference* (p. 1877).
6. Backwell, L. R., d'Errico, F., Banks, W. E., de la Peña, P., Sievers, C., Stratford, D., ... & Wadley, L., 2018. New excavations at border cave, KwaZulu-natal, South Africa. *Journal of Field Archaeology*, *43*(6), 417-436.
7. Clark, R. N., King, T. V., Klejwa, M., Swayze, G. A., & Vergo, N., 1990. High spectral resolution reflectance spectroscopy of minerals. *Journal of Geophysical Research: Solid Earth*, *95*(B8), 12653-12680.
8. Vogel, H., Meyer-Jacob, C., Thöle, L., Lippold, J.A. and Jaccard, S.L., 2016. Quantification of biogenic silica by means of Fourier transform infrared spectroscopy (FTIRS) in marine sediments. *Limnology and oceanography: methods*, *14*(12), pp.828-838.
9. Hauff, P., 2008. An overview of VIS-NIR-SWIR field spectroscopy as applied to precious metals exploration. *Spectral International Inc*, *80001*, pp.303-403.
10. Canet, C., Hernández-Cruz, B., Jiménez-Franco, A., Pi, T., Peláez, B., Villanueva-Estrada, R.E., Alfonso, P., González-Partida, E. and Salinas, S., 2015. Combining ammonium mapping and short-wave infrared (SWIR) reflectance spectroscopy to constrain a model of hydrothermal alteration for the Acoculco geothermal zone, Eastern Mexico. *Geothermics*, *53*, pp.154-165.
11. Calvin, W.M. and Pace, E.L., 2016. Mapping alteration in geothermal drill core using a field portable spectroradiometer. Geothermics, 61, pp.12-23.
12. Müller, C. M., Pejcic, B., Esteban, L., Delle Piane, C., Raven, M., & Mizaikoff, B., 2014. Infrared attenuated total reflectance spectroscopy: an innovative strategy for analyzing mineral components in energy relevant systems. *Scientific reports*, *4*(1), 1-11.
13. Preston, L. J., Melim, L. A., Polyak, V. J., Asmerom, Y., & Southam, G., 2014. Infrared spectroscopic biosignatures from Hidden Cave, New Mexico: possible applications for remote life detection. *Geomicrobiology Journal*, *31*(10), 929-941.
14. Al Omari, M.M.H., Rashid, I.S., Qinna, N.A., Jaber, A.M. and Badwan, A.A., 2016. Calcium carbonate. In *Profiles of drug substances, excipients and related methodology* (Vol. 41, pp. 31-132). Academic Press.
